# Natural-like Chalcones with Antitumor Activity on Human MG63 Osteosarcoma Cells

**DOI:** 10.3390/molecules27123751

**Published:** 2022-06-11

**Authors:** Martina Rossi, Concettina Cappadone, Giovanna Picone, Alessandra Bisi, Giovanna Farruggia, Federica Belluti, Paolo Blasi, Silvia Gobbi, Emil Malucelli

**Affiliations:** 1 Department of Pharmacy and Biotechnology, Alma Mater Studiorum-University of Bologna, Via San Donato 19/2, 40127 Bologna, Italy; martina.rossi12@unibo.it (M.R.); concettina.cappadone@unibo.it (C.C.); giovanna.picone2@unibo.it (G.P.); giovanna.farruggia@unibo.it (G.F.); p.blasi@unibo.it (P.B.); emil.malucelli@unibo.it (E.M.); 2 Department of Pharmacy and Biotechnology, Alma Mater Studiorum-University of Bologna, Via Belmeloro 6, 40126 Bologna, Italy; alessandra.bisi@unibo.it (A.B.); federica.belluti@unibo.it (F.B.)

**Keywords:** Licochalcone A, drug screening, Quantitative Phase Imaging, natural products, human fibroblasts

## Abstract

Osteosarcoma (OS) is a malignant disease characterized by poor prognosis due to a high incidence of metastasis and chemoresistance. Recently, Licochalcone A (Lic-A) has been reported as a promising agent against OS. Starting from chalcones selected from a wide in-house library, a new series was designed and synthetized. The antitumor activity of the compounds was tested on the MG63 OS cell line through the innovative Quantitative Phase Imaging technique and MTT assay. To further investigate the biological profile of active derivatives, cell cycle progression and apoptosis induction were evaluated. An earlier and more consistent arrest in the G2-M phase with respect to Lic-A was observed. Moreover, apoptosis was assessed by Annexin V staining as well as by the detection of typical morphological features of apoptotic cells. Among the selected compounds, **1e**, **1q,** and **1r** proved to be the most promising antitumor molecules. This study pointed out that an integrated methodological approach may constitute a valuable platform for the rapid screening of large series of compounds.

## 1. Introduction

Osteosarcoma (OS) is a malignant sarcoma characterized by the formation of immature bone, which is associated with a poor prognosis due to the high incidence of metastasis and frequent chemoresistance. OS is a heterogenous disease and is subclassified according to histology and the grade of malignancy as low-grade and high-grade, with the latter representing 75% of total OS cases [1,2]. While the prognosis for low-grade OS is usually favorable and most patients are mainly treated with surgery, long-term clinical outcomes of high-grade OS are much less satisfactory [3]. Indeed, high-grade OS is a malignant aggressive primary bone tumor that commonly arises in the long bones of children and adolescents [4]. Its standard clinical treatment is surgical tumor resection combined with chemotherapy protocols, generally based on “classical” anticancer compounds, such as doxorubicin, methotrexate, cisplatin, and ifosfamide [1,5]. Despite efforts made by clinicians in the last 30 years, the 5-year survival rate of high-grade OS is limited to 70%. The remaining 30% patients are not responsive to treatments, probably due to an incomplete understanding of OS pathogenesis, progression, and chemoresistance [2]. Therefore, the development of innovative and more selective strategies able to improve the survival of OS patients is a compelling unmet need [6].

Natural products (NPs) have been a source of remedies for human and animal diseases from the dawn of time. For many centuries, human beings’ health relied on empirical medicinal products of natural origin (i.e., plant, animal, and mineral), and only in the last century did drug discovery and development become an interdisciplinary science-based research field [7]. Although in the last 30 years the pharmaceutical companies demonstrated a reduced interest in NPs, NPs still maintain a prominent role as a source of new drugs [8]. Since 1981, 36.3% of the approved small molecule drugs have been directly or indirectly related to NPs. Figures are even much more convincing when the analysis is restricted to anticancer agents. Indeed, in the timeframe 1946 to 2019, 79% of the 259 approved small molecule anticancer drugs were NPs or related compounds [9], confirming their value in the search for bioactive compounds and scaffolds for the development of antitumor agents.

**Figure 1:**
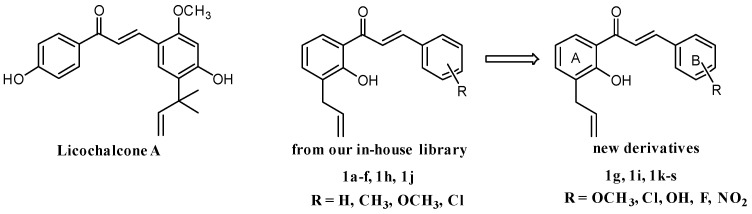
Design of the studied compounds.

Within this framework, chalcones, the precursors of flavonoids, are the perfect example of NPs that have been demonstrated to possess a wide range of promising biological activities (e.g., anti-inflammatory, antidiabetic, antimicrobial, neuroprotective, and anticancer). Different derivatives are under investigation in clinical trials, and two of them are already employed in clinics (i.e., metochalcone and sofalcone) [10,11,12]. Chalcones are widely recognized as privileged structures, families of molecules featuring scaffolds that upon appropriate decoration can lead to a large spectrum of diverse biological effects. Moreover, their synthetic accessibility makes them valuable core structures to be easily and purposely modified to obtain synthetic derivatives endowed with improved biological profiles. Licochalcone A (Lic-A, Figure 1), a natural chalcone extracted from the roots of *Glycyrrhiza glabra*, has proven to possess an intriguing biological profile, including antioxidant [13,14], antitumor [15], antimetastatic, and autophagy/apoptosis-inducing properties [16]. In particular, its ability to interfere with the growth of OS cells has been recently reported [17,18], turning the spotlight on the chalcone structure as a potential useful scaffold for the development of novel focused molecules.

The focus of this study was the identification of an easily affordable natural-like versatile synthetic scaffold that can be simply and extensively modified to open new perspectives in the therapy of OS. Due to our long-lasting drug discovery research and expertise on flavonoid-related molecules and based on the above-mentioned recent findings indicating Lic-A as an interesting chalcone active on OS cells, our in-house library of previously synthesized chalcones was visually inspected to identify molecules that could bear some similarity to Lic-A to be developed into potential antitumor agents for OS. Some compounds were retrieved, which could be somewhat related to Lic-A (**1a**–**f**, **1h**, and **1j**) (Figure 1 and Table 1), and their cytotoxicity on the MG63 human OS cell line was tested. The selected chalcones proved to inhibit the growth of OS cells to some extent and, following the encouraging results obtained, these hits were considered as a starting point to design a systematic structure–activity relationship (SAR) study to gain insight into the structural features required to improve the activity of this set of molecules. In particular, in a first optimization attempt, new derivatives were synthesized in which ring B was appropriately decorated by inserting substituents with different electronic and steric properties, and their positioning on the aromatic core was also varied to obtain the compounds **1g**, **1i**, **and 1k**–**s** (Figure 1 and Table 1). The whole series of compounds **1a**–**s** and Lic-A, included as a reference compound due to its reported activity on the same cell line, were then extensively studied to define their biological profiles. 

## 2. Results

### 2.1. Chemistry

**Scheme 1:**
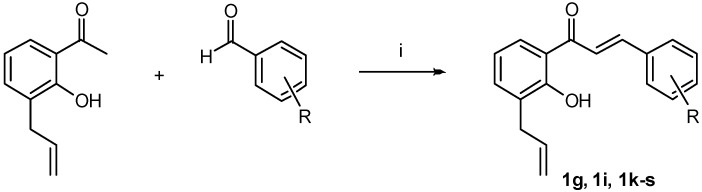
Synthesis of compounds **1g**, **1i**, and **1k**–**s**. Reagents and conditions: (i) 50% KOH, EtOH, rt, 18 h (Method A) or 2N KOH, EtOH, rt, 3 days (Method B).

The synthesis of chalcones **1a**–**f**, **1h,** and **1j** was reported in a previous paper [19]. For the synthesis of the new compounds **1g**, **1i,** and **1k**–**s**, the key intermediate 2′-hydroxy-3′-allylacetophenone [20] was reacted with the appropriately substituted benzaldehyde in EtOH and 50% KOH solution for 18 h (Method A) to give the desired chalcones (Scheme 1). For compound **1q,** the conditions were slightly modified, due to very poor yields, by using 2N KOH for 3 days (Method B).

### 2.2. Effects on MG63 Cell Proliferation

The antiproliferative activities of the studied chalcones were tested on MG63 cells by means of Quantitative Phase Imaging (QPI) technology. This innovative analytical approach allows to investigate the activity of antiproliferative agents on cells, both morphologically and quantitatively. It is a label-free methodology that is able to provide a large amount of accurate and precise data in a short time frame. 

**Figure 2:**
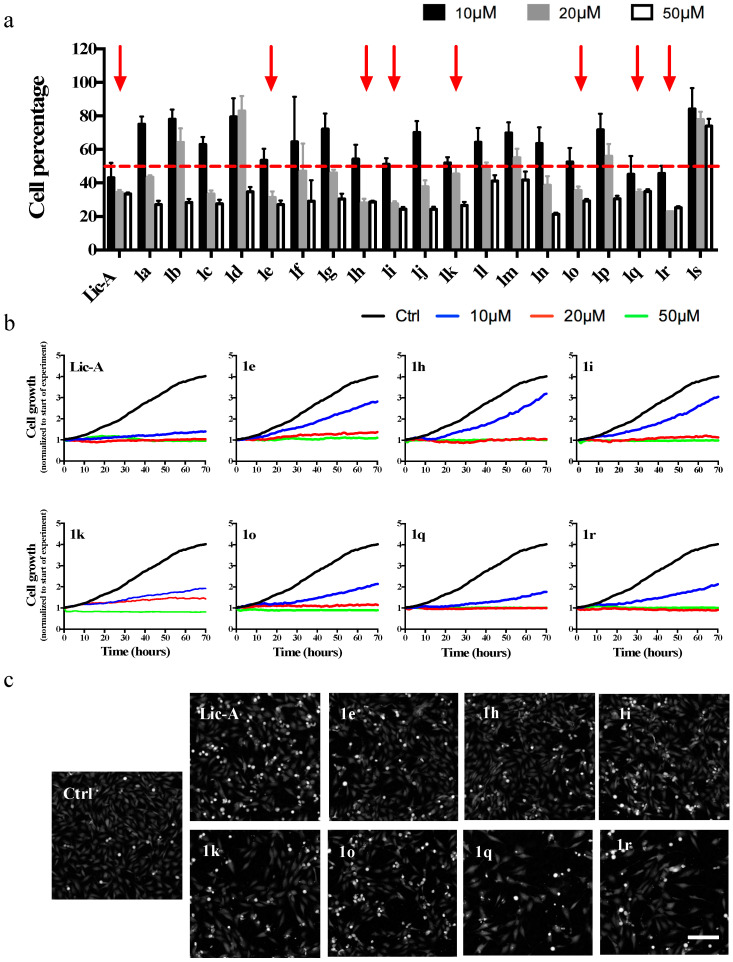
**Effectsof chalcones on MG63 cell proliferation and cell death.** Cells were incubated with 10, 20, and 50 µM of each compound and analyzed during the following 72 h. (**a**) Number of cells, expressed as percentage with respect to the control, obtained by QPI technology at 48 h. (**b**) Cell growth profiles reported as cell number fold increase, along the 72 h of incubation with the most active compounds. Data were normalized for the effective number present at the beginning of experiment. (**c**) Representative quantitative phase images recorded at 48 h after treatment with the selected derivatives at 10 µM concentration. Scale bar 200 µm.

The treatment of MG63 cells with the reference compound Lic-A at 10 µM reduced the cell number to ~40% with respect to the control within 48 h. At the highest concentrations (i.e., 20 and 50 µM), the number of cells was ~34%, without significant differences between the two concentrations (Figure 2a). Among the investigated nineteen compounds (Figure 1), seven emerged (i.e., **1e**, **1h**, **1i**, **1k**, **1o**, **1q,** and **1r**) since they showed effects comparable to those induced by Lic-A (Table 1 and Figure 2a). All these compounds halved the cell population already at the lowest tested concentration (10 µM), with more consistent effects at the higher ones. The antiproliferative effects induced by these compounds were dose-dependent and persistent in time (Figure 2b). Considering that the chalcone structure is characterized by an α,β-unsaturated carbonyl function, a well-known Michael acceptor group, which may be responsible for covalent binding to thiol moieties, covalent modifications of potentially involved molecular targets cannot be excluded.

The antitumor activity was confirmed by the reduced cell density and by the presence of many floating cells after treatment with the selected compounds (Figure 2c). Notably, these effects were more evident with compounds **1q** and **1r**, both decorated with a nitro group [21]. In addition, at 48 h, some distinctive features of apoptotic cells, such as membrane blebs or nuclear fragmentation, were detected. 

### 2.3. Effects on MG63 Cell Viability and Metabolic Activity

Mitochondrial metabolism for the whole cell population was evaluated by an MTT assay. This method is commonly used as an indicator of cell viability, also allowing to calculate IC_50_ values. Interestingly, the previously applied QPI technology permits to count and analyze each single cell, monitoring its behavior in time. Single-cell analysis is a fast-growing field with a high impact in the biomedical field, with interesting applications in cancer research, diagnostics, and drug discovery. Therefore, information provided by single-cell and large population analyses are complementary, and it is advisable to exploit the opportunity to merge, when possible, the two approaches [22]. 

**Figure 3:**
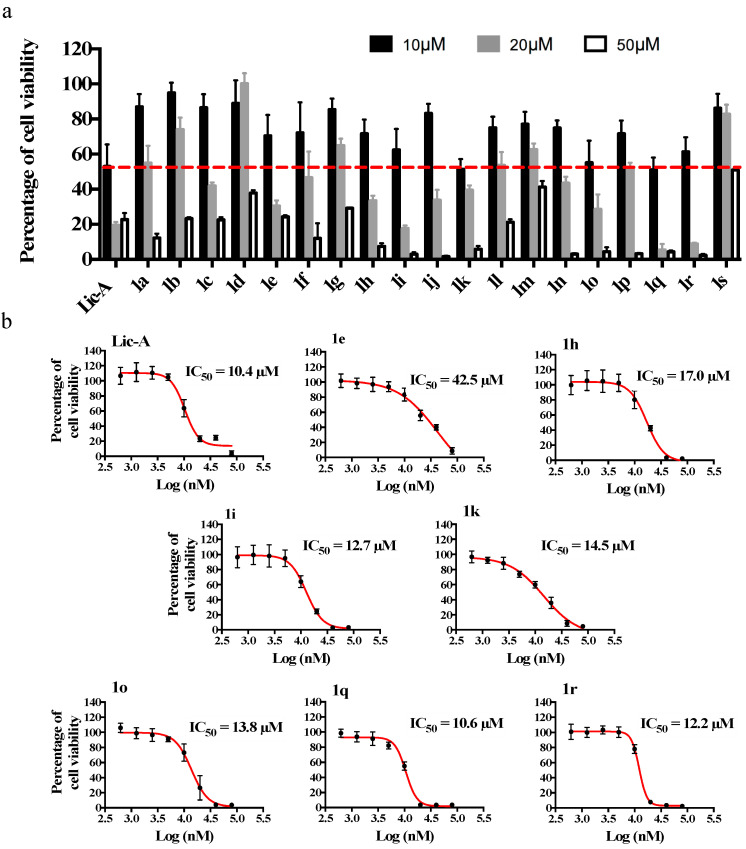
**Effects of chalcones on MG63 cell viability by MTT assay 48 h after treatment**. (**a**) Cells were incubated with 10, 20, and 50 µM of each compound; viable cells are expressed as percentage with respect to the control. (**b**) Dose–response curves and IC_50_ calculation. Cells were incubated with 80, 40, 20, 10, 5, 2.5, 1.25, and 0.625 µM of Lic-A or the selected compounds.

In this perspective, cytotoxic effects of the studied chalcones and Lic-A were investigated through MTT assay after 48 h of treatment. Figure 3a shows that treatment with 10 µM Lic-A reduced cell viability to ~50%. In addition, both the 20 and 50 µM concentrations induced a further decrease in cell population up to ~80%, similarly to what was observed with the QPI analysis (Figure 2a). Again, the seven chalcones (i.e., **1e**, **1h**, **1i**, **1k**, **1o**, **1q,** and **1r**) proved to be the most potent compounds. In particular, the metabolic activity reduction induced by **1i**, **1k**, **1o**, **1q,** and **1r** was comparable to Lic-A at 10 µM, while **1e** and **1h** showed consistent effects only at higher concentrations. Figure 3b depicts the time–response curves for the selected compounds. All the IC_50_ values ranged from 10 to 20 µM, except for **1e**, showing an IC_50_ of 42.5 µM. It is worth noting that the dose–response curves for **1q** and **1r** showed a steep slope, whereas the others, in particular those of **1e** and **1k,** exhibited a shallow one. It is well known that a course of the dose–response curve that is less steep indicates a greater chance of a finer activity control, since around the IC_50_ a small increase in concentration does not determine maximum toxicity. On the contrary, the steep curve obtained for **1q** and **1r**, both carrying a nitro group, could indicate potential toxic side effects. Indeed, although regarded as a versatile functional group in medicinal chemistry, this moiety may bring about toxicity issues, being often considered a toxicophore, mainly due to its enzymatic reduction products. On the other hand, previously neglected nitro-containing drugs are lately being reconsidered for drug repositioning programs [21].

Taking into account the data obtained with the two different biological assays (i.e., QPI and MTT assays), compounds **1e**, **1h**, **1i**, **1k**, **1o**, **1q,** and **1r** were chosen for in-depth evaluation. 

### 2.4. Analysis of Cytotoxicity on Normal Human Fibroblasts

It is well known that antitumor drugs primarily act on fast-growing cells. Therefore, a major obstacle related to this therapeutic approach lies in its poor selectivity, so that rapidly dividing and growing healthy cells are affected as well, often resulting in the failure of conventional chemotherapy due to side effects. Moreover, high doses of drugs are often required to reach the tumor microenvironment, consequently leading to nonspecific toxicity [23]. 

**Figure 4:**
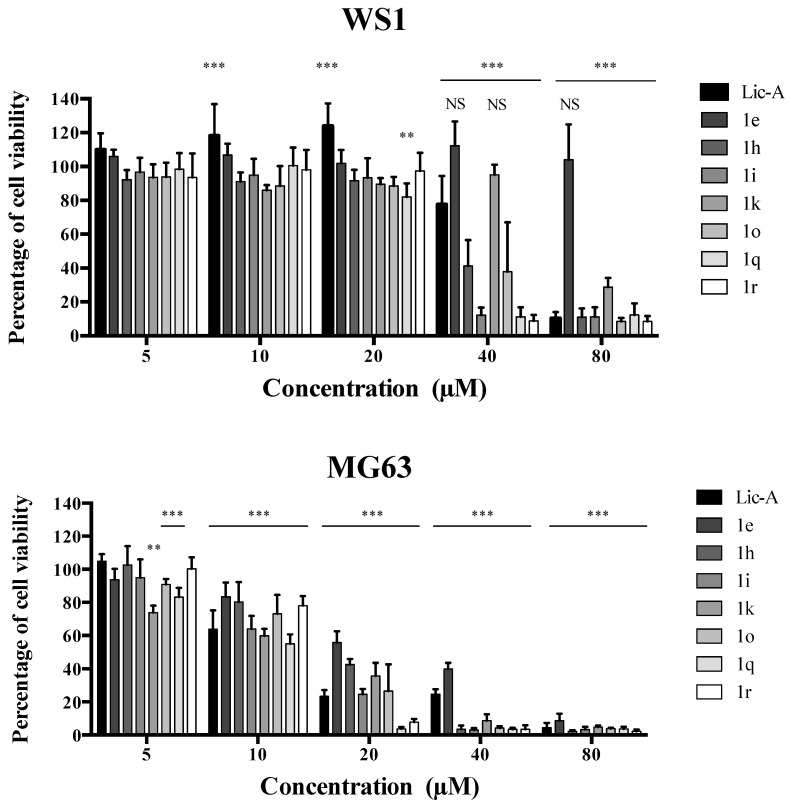
**Comparative analysis of chalcone effects on cell viability in WS1 normal human fibroblasts and MG63 cells.** Cells were treated with five different concentrations (80, 40, 20, 10, and 5 µM). MTT assay was performed 48 h after treatment. Two-way ANOVA was performed, and all treatments were analyzed against control, ** *p* < 0.01, *** *p* < 0.001, and NS not statistically significant. Note that the data for MG63 are reported from Figure 3 and shown only for comparison.

To assess the selectivity of the selected chalcones toward cancer cells and to rule out nonspecific toxicity, their activities were tested on the WS1 normal human fibroblast cell line. This experimental model was chosen as fibroblasts are present in connective tissue and synthetize collagen and other extracellular matrix proteins. These characteristics make this model suitable to evaluate the selectivity of new compounds against OS [24]. The effects on cell viability were investigated by the MTT assay, and Lic-A was also tested for comparison (Figure 4). 

Unlike the results obtained in cancer cells, all the molecules were able to induce cytotoxic effects in healthy cells only at 40 and 80 µM. Among the tested compounds, only **1q** showed a moderate reduction of cell viability at 20 µM (82.1 ± 7.8%), even though it maintained a remarkable selectivity toward MG63 cells (Figure 4). Therefore, considering their selectivity, the studied compounds can be considered promising tools to contrast OS cell proliferation. 

### 2.5. Effects on MG63 Cell Cycle

**Figure 5:**
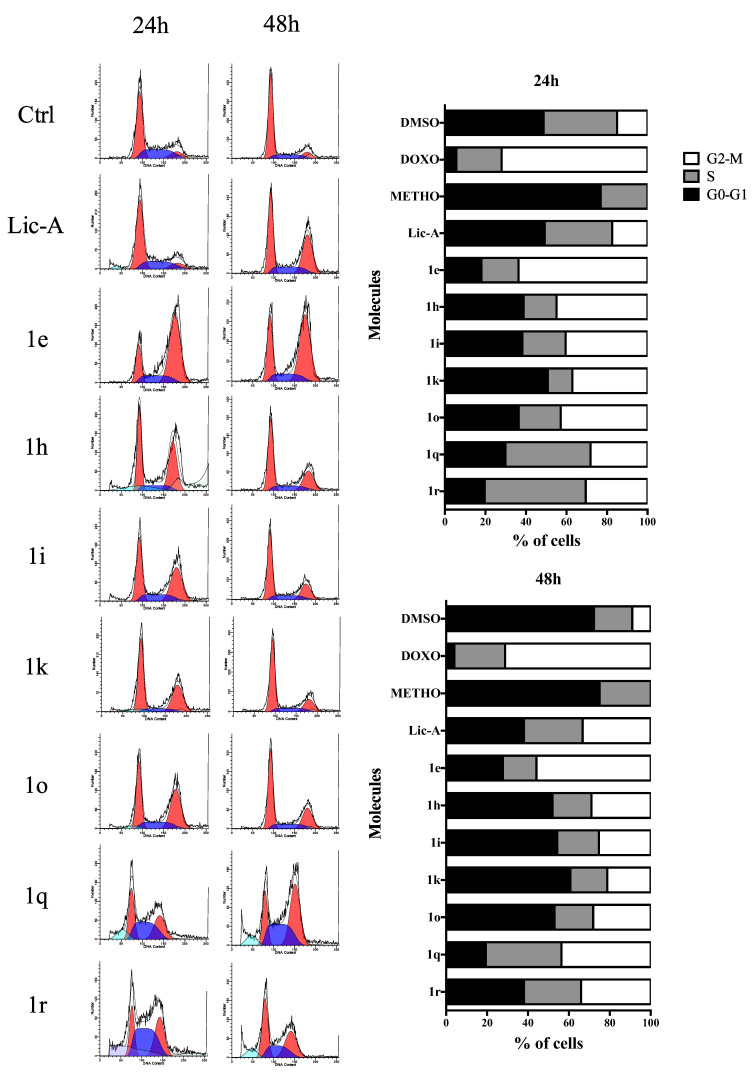
**Cell cycle analyses.** Cell cycle analyses were performed after 24 or 48 h of treatment with the studied compounds and Lic-A at IC_50_ concentrations. (**a**) The histogram shows the cell cycle profile of a representative experiment. (**b**) Percentage distribution of cells in the different cell cycle phases.

To clarify the activity of the selected derivatives on cell cycle progression, cell distribution into the different phases was investigated. Cell cycle profiles of MG63 cells treated with the selected compounds, and Lic-A as reference, at their IC_50_s were obtained by flow cytometry. As the positive control, OS cells were treated with two commonly employed anticancer drugs inducing different effects on the cell cycle. In particular, doxorubicin is able to induce G2-M phase arrest, while methotrexate causes an accumulation in G0-G1 (Figure S1). As reported in Figure 5, the effects induced on the cell cycle by Lic-A (G2-M arrest) can be clearly appreciated at 48 h, while in our assay conditions, only minimal alterations were observed at 24 h. These results are in accordance with recent published data by Shen et al. on a human OS cell model [17]. The results obtained for the molecules under study proved to be more similar to those obtained with doxorubicin (G2-M arrest) rather than methotrexate (G0-G1 arrest). Interestingly, all the new compounds induced a remarkable block in G2-M, higher than Lic-A, which showed 17.5% of cells in this phase. At 24 h, compound **1e** proved to be the most potent, showing a value of 63.6%, while a range between 28% (**1q**) and 45% (**1h**) was observed for the other derivatives. Looking at the results at 48 h, the G2-M arrest was still evident for all compounds, in particular for **1e, 1q,** and **1r**. It is important to note a pre-G1 peak in the DNA profile for **1q** and **1r**, possibly indicating that the antiproliferative effect culminates in apoptosis. These results agree with data previously obtained by QPI analysis, showing morphological characteristics of apoptotic cell death. 

### 2.6. Effects on MG63 Apoptosis: Phosphatidylserine Exposure

**Figure 6:**
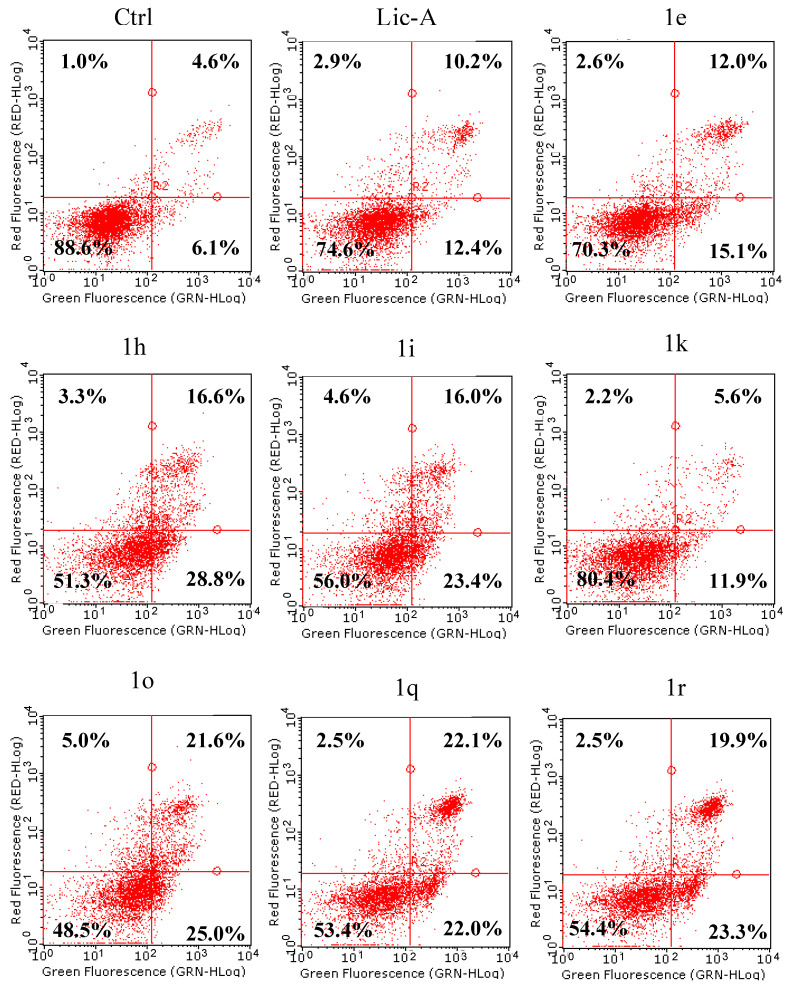
**Annexin V and Propidium staining.** Cytofluorimetric analysis of annexin V-FITC- and PI-stained cells after 16h treatment with compounds at IC_50_ concentrations. At least 5000 cells per sample were analyzed, and quadrant analysis was performed. Left quadrant corresponds to the double negative cells; lower right to the Annexin V-positive and PI-negative cells (early apoptotic); upper right to the double-positive cells (late apoptotic/necrotic); and upper left to the PI-positive and Annexin V-negative cells (necrotic).

To further investigate the effects of the selected chalcones on cell death and to elucidate if they could be regarded as apoptotic inducers, MG63 cells were stained after 16 h of treatment with annexin V and propidium iodide (PI). The affinity of the cells for annexin V indicates that these cells have exposed phosphatidylserine on their outer membrane leaflet, a typical event triggering apoptosis [25], while PI staining indicates alteration in membrane integrity, which occurs in necrosis or in late apoptosis. The results showed a general increase in cells labeled with annexin V as the marker of early apoptosis (Figure 6). In particular, cells treated with **1e** and **1k** exhibited 15.1% and 11.9% of apoptotic cells, respectively, similar to Lic-A (12.4%). Cells treated with **1h**, **1i**, **1o**, **1q,** and **1r** showed a percentage of about 25% with respect to the 6.1% of control cells. To obtain a more complete picture of the apoptotic effect, the percentage of cells defined as late apoptotic/necrotic (double positive for Annexin V and PI) should be taken into account. For the most potent compounds, the late apoptotic cells were around 20%. Notably, **1h**, **1i**, **1o**, **1q,** and **1r**, all substituted with electron-withdrawing groups, showed the highest percentage of apoptotic cells (early plus late).

### 2.7. Effects on MG63 Apoptosis: Cytoskeleton Changes and Nuclear Morphology

The actin cytoskeleton is a structural network of proteins that are essential for multiple biological functions, including cell contraction, cell motility, vesicle trafficking, endocytosis, and apoptosis [26]. It has been demonstrated to be modified in apoptosis, and changes in its organization are associated with different stages of this process. The degradation of actin cytoskeleton before the phagocytosis of the apoptotic bodies has also been largely reported. Additionally, the role of actin among the morphological hallmarks of apoptosis is supported by mounting evidence depicting it as a mediator and initiator of apoptosis signaling [27].

**Figure 7:**
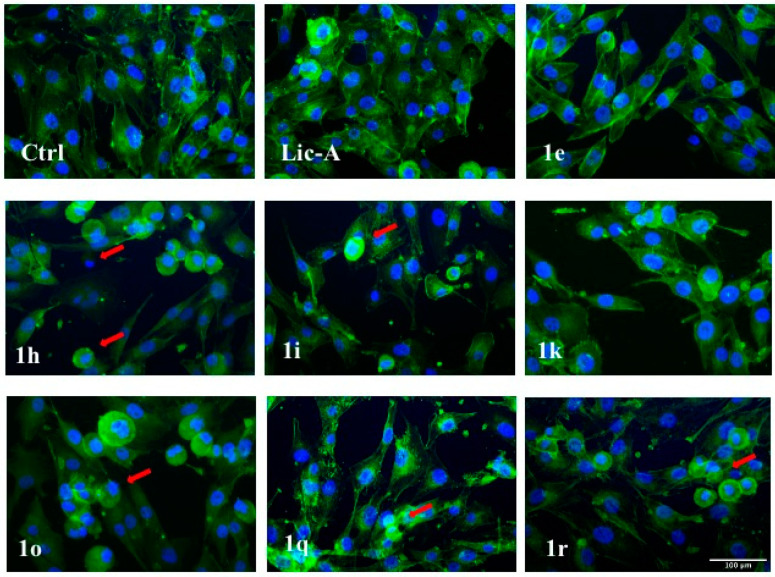
**Effects of chalcones on MG63 morphology.** Fluorescence microscopy of control and cells treated at IC_50_ concentrations for 24 h. Nuclei and F-actin are stained with Hoechst (blue) and phalloidin (green) respectively. The arrows indicated the destructured F-actin fibers. Scale bar 100 μm.

To verify the apoptotic effects of the selected chalcones on MG63 morphology, nuclei and cytoskeleton were studied by immunofluorescence. In detail, nuclei were stained with Hoechst (blue) and F-actin filaments with phalloidin-FITC (green). At 24 h, cells displayed evident nuclei fragmentation, membrane blebbing, and stretched filamentous F-actin strings, which became shorter and irregularly destructured (Figure 7). All these morphological alterations are consistent with the induction of apoptosis [26,27]. Furthermore, in line with the G2-M arrest previously reported, in most treatments, several cells with a rounded shape and condensed chromatin were observed (see Figure S2). This evidence suggested that treatments induced arrest of cell cycle progression in M rather than in the G2 phase. The ability of these compounds to block cell mitosis makes them worthy of further studies and could drive the research toward the investigation of cell signaling pathways and molecular targets involved in the activity of other antimitotic drugs. Again, **1h**, **1i**, **1o**, **1q,** and **1r** seemed to be the most potent derivatives. In particular, the cytoskeleton of cells treated with **1q** and **1r** appeared disassembled, while the nuclei in cells treated with **1h** were fragmented.

## 3. Materials and Methods

### 3.1. Chemistry

#### 3.1.1. General Methods

Starting materials, unless otherwise specified, were used as high-grade purity commercial products. Solvents were of analytical grade. Reaction progress was followed by thin layer chromatography (TLC) on precoated silica gel plates (Merck Silica Gel 60 F254) and then visualized with a UV254 lamplight. Chromatographic separations were performed on silica gel columns by flash method (Kieselgel 40, 0.040–0.063 mm, Merck). Melting points were determined in open glass capillaries using a Büchi apparatus and were uncorrected. ^1^H NMR and ^13^C NMR spectra were recorded on a Varian Gemini spectrometer 400 MHz and 101 MHz, respectively, in CDCl_3_ solutions, and chemical shifts (δ) were reported as parts per million (ppm) values relative to tetramethylsilane (TMS) as the internal standard; coupling constants (*J*) were reported in Hertz (Hz). Standard abbreviations indicating spin multiplicities are given as follow: s (singlet), d (doublet), t (triplet), br (broad), q (quartet), or m (multiplet). UHPLC−MS analyses were run on a Waters ACQUITY ARC UHPLC/MS system consisting of a QDA mass spectrometer equipped with an electrospray ionization interface and a 2489 UV/Vis detector. The detected wavelengths (λ) were 254 nm and 365 nm. The analyses were performed on an XBridge BEH C18 column (10 × 2.1 mm i.d., particle size 2.5 μm) with an XBridge BEH C18 VanGuard Cartridge precolumn (5 mm × 2.1 mm i.d., particle size 1.8 μm). The mobile phases were H_2_O (0.1% formic acid) (A) and MeCN (0.1% formic acid) (B). Linear gradient: 0−0.78 min, 20% B; 0.78–2.87 min, 20−95% B; 2.87–3.54 min, 95% B; 3.54–3.65 min, 95–20% B; and 3.65–5.73, 20% B. Flow rate: 0.8 mL/min. Electrospray ionization in positive and negative modes was applied in the mass scan range 50−1200 Da. All tested compounds were found to have >95% purity. High resolution mass spectra were recorded on Waters Xevo G2-XS QTof apparatus operating in electrospray mode (ES). Compounds were named relying on the naming algorithm developed by CambridgeSoft Corporation (Cambridge, Massachusetts, US) and used in ChemDraw Professional 19.0.

#### 3.1.2. General Procedures for the Synthesis of Chalcones **1g**, **1i**, **1k**–**s**

Method A: 50% KOH solution (0.4 mL/mmol) was added dropwise to a mixture of 2′-hydroxy-3′-allylacetophenone [20] (1 eq) and the selected benzaldehyde (1 eq) in EtOH (3–4 mL/mmol), and the mixture was stirred at rt overnight. The reaction mixture was then poured into ice and acidified with HCl. The precipitate was filtered (or the mixture was extracted with EA), and the crude was purified by flash chromatography (petroleum ether/toluene 4:1 or 1:1 or petroleum ether/ethyl acetate 4:1 or 9:1) and/or crystallized from MeOH.

Method B: 2N KOH solution (0.4 mL/mmol) was added dropwise to a mixture of 2′-hydroxy-3′-allylacetophenone [20] (1 eq) and the selected benzaldehyde (1 eq) in EtOH (3–4 mL/mmol), and the mixture was stirred at rt for 3–4 days. The reaction mixture was then poured into ice and acidified with HCl. The precipitate was filtered (or the mixture was extracted with EA), and the crude was purified by flash chromatography (petroleum ether/toluene 4:1 or 1:1) and/or crystallized from MeOH.

##### (E)-1-(3-Allyl-2-hydroxyphenyl)-3-(4-methoxyphenyl)prop-2-en-1-one (**1g**)

Starting from 4-methoxybenzaldehyde (150 mg, 1.1 mmol) and following method A, 35 mg (yield 10%) of **1g** were obtained as yellow solid, mp 83–84 °C. ^1^H NMR: δ 13.30 (s, 1H, OH), 7.91 (d, *J* = 15.4 Hz, 1H, COCH=), 7.82 (d, *J* = 8.1 Hz, 1H, arom), 7.64 (d, *J* = 8.7 Hz, 2H, arom), 7.56 (d, *J* = 15.4 Hz, 1H, CH=), 7.38 (d, *J* = 7.2 Hz, 1H, arom), 6.96 (d, *J* = 8.7 Hz, 2H, arom), 6.90 (t, *J* = 7.7 Hz, 1H, arom), 6.05 (ddt, *J* = 16.8, 10.2, 6.6 Hz, 1H, CH=), 5.21 –4.99 (m, 2H, CH_2_=), 3.88 (s, 3H, CH_3_), and 3.48 (d, *J* = 6.6 Hz, 2H, CH_2_). ^13^C NMR: δ 193.9, 162.0, 161.6, 145.1, 136.2, 136.1, 130.5 (2C), 129.5, 127.6, 127.4, 119.6, 118.2, 117.9, 115.9, 114.5 (2C), 55.4, and 33.5. HRMS C_19_H_18_O_3_ [M+H] calculated: 295.1334, found: 295.1327.

##### (E)-1-(3-Allyl-2-hydroxyphenyl)-3-(3-chlorophenyl)prop-2-en-1-one (**1i**)

Starting from 3-chlorobenzaldehyde (154 mg, 1.1 mmol) and following method A, 30 mg (yield 9%) of **1i** were obtained as yellow solid, mp 58–59 °C. ^1^H NMR: δ 13.08 (s, 1H, OH), 7.88–7.79 (m, 2H, arom), 7.70–7.63 (m, 2H, arom), 7.53 (dt, *J* = 7.0, 1.7 Hz, 1H, arom), 7.45–7.35 (m, 3H, arom), 6.92 (t, *J* = 7.7 Hz, 1H, arom), 6.05 (ddt, *J* = 16.9, 10.2, 6.6 Hz, 1H, CH=), 5.17–5.06 (m, 2H, CH_2_=), and 3.48 (dd, *J* = 6.6, 1.6 Hz, 2H, CH_2_). ^13^C NMR: δ 193.6, 161.7, 143.5, 136.7, 136.5, 136.1, 135.1, 130.6, 130.3, 129.7, 128.0, 127.8, 127.0, 121.7, 119.4, 118.5, 116.1, and 33.5. HRMS C_18_H_15_ClO_2_ [M+H] calculated: 299.0839, found: 299.0836.

##### (E)-1-(3-Allyl-2-hydroxyphenyl)-3-(2-hydroxyphenyl)prop-2-en-1-one (**1k**)

Starting from 2-hydroxybenzaldehyde (134 mg, 1.1 mmol) and following method A, 20 mg (yield 6.5%) of **1k** were obtained as yellow solid, mp 150–151 °C. ^1^H NMR: δ 13.26 (s, 1H, OH), 8.16 (d, *J* = 15.6 Hz, 1H, COCH=), 7.92–7.79 (m, 2H, arom), 7.61 (d, *J* = 7.7 Hz, 1H, arom), 7.39 (d, *J* = 7.2 Hz, 1H, arom), 7.31 (d, *J* = 7.8 Hz, 1H, arom), 7.01 (t, *J* = 7.6 Hz, 1H, arom), 6.95–6.83 (m, 2H, arom), 6.05 (td, *J* = 17.0, 6.7 Hz, 1H, CH=), 5.59 (br, 1H, OH), 5.18–5.05 (m, 2H, CH_2_=), and 3.48 (d, *J* = 6.5 Hz, 2H, CH_2_). ^13^C NMR: δ 194.5, 161.6, 155.4, 140.6, 137.3, 136.3, 136.2, 131.9, 130.2, 129.5, 127.9, 121.6, 121.3, 119.6, 118.3, 116.5, 116.0, and 33.5. HRMS C_18_H_16_O_3_ [M+H] calculated: 281.1177, found: 281.1174.

##### (E)-1-(3-Allyl-2-hydroxyphenyl)-3-(3-hydroxyphenyl)prop-2-en-1-one (**1l**)

Starting from 3-hydroxybenzaldehyde (134 mg, 1.1 mmol) and following method A, 61 mg (yield 19.8%) of **1l** were obtained as yellow solid, mp 113–114 °C. ^1^H NMR: δ 13.15 (d, *J* = 0.6 Hz, 1H, OH), 7.86 (d, *J* = 15.5 Hz, 1H, CO CH=), 7.81 (dd, *J* = 8.2, 1.6 Hz, 1H, arom), 7.69–7.61 (m, 1H, arom), 7.43–7.38 (m, 1H, arom), 7.32 (t, *J* = 7.8 Hz, 1H, arom), 7.25 (dt, *J* = 7.7, 1.5 Hz, 1H, arom), 7.16–7.12 (m, 1H, arom), 6.95–6.86 (m, 2H, arom), 6.05 (ddt, *J* = 16.8, 10.2, 6.6 Hz, 1H, CH=), 5.16–5.07 (m, 2H, CH_2_=), 4.92 (br, 1H, OH), and 3.48 (dt, *J* = 6.6, 1.6 Hz, 2H, CH_2_). ^13^C NMR: δ 193.9, 161.6, 156.0, 144.8, 136.5, 136.3, 136.1, 130.3, 129.6, 127.8, 121.6, 120.9, 119.5, 118.4, 118.0, 116.0, 114.8, and 33.5. HRMS C_18_H_16_O_3_ [M+H] calculated: 281.1177, found: 281.1169.

##### (E)-1-(3-Allyl-2-hydroxyphenyl)-3-(4-hydroxyphenyl)prop-2-en-1-one (**1m**)

Starting from 4-hydroxybenzaldehyde (134 mg, 1.1 mmol) and following method A, 22 mg (yield 7%) of **1m** were obtained as yellow solid, mp 146–147 °C. ^1^H NMR: δ 13.27 (s, 1H, OH), 7.90 (d, *J* = 15.4 Hz, 1H, COCH=), 7.82 (d, *J* = 6.9 Hz, 1H, arom), 7.63–7.52 (m, 3H, arom), 7.39 (d, *J* = 7.4 Hz, 1H, arom), 6.89 (dd, *J* = 8.1, 6.5 Hz, 3H, arom), 6.05 (ddt, *J* = 16.8, 10.2, 6.6 Hz, 1H, CH=), 5.18–5.05 (m, 3H, CH_2_= + OH), and 3.48 (d, *J* = 6.6 Hz, 2H, CH_2_). ^13^C NMR: δ 193.9, 161.6, 158.2, 145.1, 136.2, 136.2, 130.7, 130.1, 129.5, 128.5, 127.6, 127.6, 119.6, 118.3, 118.0, 116.0, 116.0, and 33.5. HRMS C_18_H_16_O_3_ [M+H] calculated: 281.1177, found: 281.1175.

##### (E)-1-(3-Allyl-2-hydroxyphenyl)-3-(2-fluorophenyl)prop-2-en-1-one (**1n**)

Starting from 2-fluorobenzaldehyde (140 mg, 1.1 mmol) and following method A, 50 mg (yield 16%) of **1n** were obtained as yellow solid, mp 75–77 °C. ^1^H NMR: δ 13.13 (s, 1H, OH), 8.01 (d, *J* = 15.7 Hz, 1H, COCH=), 7.85–7.77 (m, 2H, arom), 7.66 (td, *J* = 7.6, 1.6 Hz, 1H, arom), 7.46–7.36 (m, 2H, arom), 7.25–7.12 (m, 2H, arom), 6.91 (t, *J* = 7.7 Hz, 1H, arom), 6.05 (ddt, *J* = 16.9, 10.2, 6.6 Hz, 1H, CH=), 5.18–4.99 (m, 2H, CH_2_=), and 3.48 (d, *J* = 6.6 Hz, 2H, CH_2_). ^13^C NMR: δ 194.0, 161.8 (d, *J* = 252 Hz), 161.6, 138.0, 136.5, 136.1, 132.1 (d, *J* = 9.0 Hz), 130.1, 129.5, 127.8, 124.5 (d, *J* = 3.0 Hz), 123.1 (d, *J* = 8.0 Hz), 122.8 (d, *J* = 11 Hz), 119.4, 118.4, 116.3 (d, *J* = 21 Hz), 116.0, and 33.5. HRMS C_18_H_15_FO_2_ [M+H] calculated: 283.1134, found: 283.1130.

##### (E)-1-(3-Allyl-2-hydroxyphenyl)-3-(3-fluorophenyl)prop-2-en-1-one (**1o**)

Starting from 3-fluorobenzaldehyde (140 mg, 1.1 mmol) and following method A, 62 mg (yield 20%) of **1o** were obtained as yellow solid, mp 75–77 °C. ^1^H NMR: δ 13.08 (s, 1H, OH), 7.87 (d, *J* = 15.5 Hz, 1H, COCH=), 7.81 (dd, *J* = 8.1, 1.5 Hz, 1H, arom), 7.67 (d, *J* = 15.5 Hz, 1H, CH=), 7.45–7.35 (m, 4H, arom), 7.18–7.11 (m, 1H, arom), 6.95–6.88 (m, 1H, arom), 6.05 (ddt, *J* = 16.9, 10.3, 6.6 Hz, 1H, CH=), 5.17–5.08 (m, 2H, CH_2_=), and 3.48 (d, *J* = 6.6 Hz, 2H, CH_2_). ^13^C NMR: δ 193.7, 163.1 (d, *J* = 245 Hz), 161.7, 143.7, 136.9 (d, *J* = 8.0 Hz), 136.7, 136.1, 130.6 (d, *J* = 8.0 Hz), 129.7, 127.8, 124.7 (d, *J* = 3.0 Hz), 121.7, 119.4, 118.5, 117.7 (d, *J* = 21 Hz), 116.1, 114.6 (d, *J* = 21 Hz), and 33.5. HRMS C_18_H_15_FO_2_ [M+H] calculated: 283.1134, found: 283.1131.

##### (E)-1-(3-Allyl-2-hydroxyphenyl)-3-(4-fluorophenyl)prop-2-en-1-one (**1p**)

Starting from 4-fluorobenzaldehyde (140 mg, 1.1 mmol) and following method A, 33 mg (yield 10.6%) of **1p** were obtained as yellow solid, mp 71–72 °C. ^1^H NMR: δ 13.15 (s, 1H, OH), 7.89 (d, *J* = 15.5 Hz, 1H, COCH=), 7.84–7.79 (m, 1H, arom), 7.67 (dd, *J* = 8.6, 5.4 Hz, 2H, arom), 7.61 (d, *J* = 15.5 Hz, 1H, CH=), 7.41 (d, *J* = 6.7 Hz, 1H, arom), 7.14 (t, *J* = 8.6 Hz, 2H, arom), 6.91 (t, *J* = 7.7 Hz, 1H, arom), 6.05 (ddt, *J* = 16.9, 10.2, 6.6 Hz, 1H, CH=), 5.18–5.00 (m, 2H, CH_2_=), and 3.48 (d, *J* = 6.6 Hz, 2H, CH_2_). ^13^C NMR: δ 193.7, 164.2 (d, *J* = 251 Hz), 161.6, 143.9, 136.5, 136.1, 130.9 (d, *J* = 3.0 Hz), 130.6 (2C, d, *J* = 9.0 Hz), 129.6, 127.7, 120.1, 119.4, 118.4, 116.2 (2C, d, *J* = 21 Hz), 116.0, and 33.5. HRMS C_18_H_15_FO_2_ [M+H] calculated: 283.1134, found: 283.1130.

##### (E)-1-(3-Allyl-2-hydroxyphenyl)-3-(2-nitrophenyl)prop-2-en-1-one (**1q**)

Starting from 2-nitrobenzaldehyde (332 mg, 2.2 mmol) and following method B, 68 mg (yield 10%) of **1q** were obtained as yellow solid, mp 95–96 °C. ^1^H NMR: δ 12.89 (s, 1H, OH), 8.26 (d, *J* = 15.4 Hz, 1H, COCH=), 8.07 (dd, *J* = 8.1, 1.0 Hz, 1H, arom), 7.80–7.64 (m, 3H, arom), 7.61–7.54 (m, 1H, arom), 7.50 (d, *J* = 15.4 Hz, 1H, CH=), 7.39 (d, *J* = 7.3 Hz, 1H, arom), 6.88 (t, *J* = 7.7 Hz, 1H, arom), 6.02 (ddt, *J* = 17.0, 10.4, 6.6 Hz, 1H, CH=), 5.14–5.04 (m, 2H, CH_2_=), and 3.45 (d, *J* = 6.6 Hz, 2H, CH_2_). ^13^C NMR: δ 193.4, 161.7, 148.7, 140.4, 136.9, 136.0, 133.5, 131.2, 130.5, 129.8, 129.3, 128.0, 125.5, 125.1, 119.2, 118.5, 116.1, and 33.5. HRMS C_18_H_15_NO_4_ [M+H] calculated: 310.1079, found: 310.1076.

##### (E)-1-(3-Allyl-2-hydroxyphenyl)-3-(3-nitrophenyl)prop-2-en-1-one (**1r**)

Starting from 3-nitrobenzaldehyde (165 mg, 1.1 mmol) and following method A, 25 mg (yield 7.4%) of **1r** were obtained as yellow solid, mp 89–90 °C. ^1^H NMR: δ 12.98 (s, 1H, OH), 8.55 (t, *J* = 1.9 Hz, 1H, arom), 8.31–8.27 (m, 1H, arom), 7.97–7.91 (m, 2H, arom), 7.84 (dd, *J* = 8.1, 1.4 Hz, 1H, arom), 7.79 (d, *J* = 15.6 Hz, 1H, CH=), 7.65 (t, *J* = 8.0 Hz, 1H, arom), 7.44 (d, *J* = 6.6 Hz, 1H, arom), 6.98–6.92 (m, 1H, arom), 6.05 (ddt, *J* = 16.9, 10.3, 6.6 Hz, 1H, CH=), 5.17–5.09 (m, 2H, CH_2_=), and 3.49 (d, *J* = 6.6 Hz, 2H, CH_2_). ^13^C NMR: δ 193.2, 161.7, 148.8, 142.0, 137.0, 136.4, 136.0, 134.5, 130.1, 129.8, 127.8, 124.9, 123.3, 122.4, 119.3, 118.6, 116.1, and 33.5. HRMS C_18_H_15_NO_4_ [M+H] calculated: 310.1079, found: 310.1073.

##### (E)-1-(3-Allyl-2-hydroxyphenyl)-3-(4-nitrophenyl)prop-2-en-1-one (**1s**)

Starting from 4-nitrobenzaldehyde (317 mg, 2.1 mmol) and following method A, 34 mg (yield 5%) of **1s** were obtained as yellow solid, mp 169–172 °C. ^1^H NMR: δ 12.95 (s, 1H, OH), 8.31 (d, *J* = 8.8 Hz, 2H, arom), 7.93 (d, *J* = 15.6 Hz, 1H, COCH=), 7.85–7.75 (m, 4H, arom), 7.44 (d, *J* = 6.8 Hz, 1H, arom), 6.94 (t, *J* = 7.7 Hz, 1H, arom), 6.04 (ddt, *J* = 17.1, 10.5, 6.6 Hz, 1H, CH=), 5.16–5.08 (m, 2H, CH_2_=), and 3.49 (d, *J* = 6.6 Hz, 2H, CH_2_). ^13^C NMR: δ 193.2, 161.8, 148.7, 141.9, 140.8, 137.1, 135.9, 129.9, 129.1 (2C), 127.8, 124.4, 124.3 (2C), 119.3, 118.6, 116.2, and 33.5. HRMS C_18_H_15_NO_4_ [M+H] calculated: 310.1079, found: 310.1071.

### 3.2. Cell Culture and Treatments

The MG63 cell line was used as the OS model, while the WS1 human fibroblast cell line (American Type Culture Collection, ATCC, Manassas, VA, USA) was used as the nontumor model. Cells were grown routinely in 5% CO_2_/humidified air at 37 °C with Dulbecco’s modified Eagle medium supplemented with D-glucose (4.5 g/L), fetal bovine serum (10% *v*/*v*), L-glutamine (2 mM), penicillin (1000 U/mL), and 1 mg/mL of streptomycin (Euroclone S.p.A., Milan, Italy). Cells were seeded at 5.000 cells/well (15 × 10^3^ cell/cm^2^) in a 96-well plate (Corning^®^, Corning, NY, USA) and incubated 24 h to allow cell adhesion. After adhesion, the medium was removed and replaced with fresh medium containing the desired concentration of the compounds to be tested.

Tested compounds were dissolved in dimethyl sulfoxide (DMSO) (product reference, 276855; Sigma-Aldrich Chemie GmbH, Schnelldorf, Germany) at 50 mM to produce a stock solution that was added to the cell culture medium to achieve the final concentrations of 10, 20, and 50 µM. The vehicle, DMSO, was used as a negative control, while Lic-A (product reference, 435800; Merk KGaA, Darmstadt, Germany) (DMSO stock solution, 29.5 mM) was used as a reference compound. Doxorubicin (product reference, 44583; Sigma-Aldrich Chemie GmbH, Schnelldorf, Germany) (water stock solution, 2.76 mM) and methotrexate (product reference, M1000000; Sigma-Aldrich Chemie GmbH, Schnelldorf, Germany) (DMSO stock solution, 50 mM), commonly employed in OS chemotherapy protocols, were used as reference drugs at the following final concentrations: 640, 320, and 160 nM for doxorubicin; 80, 40, and 20 nM for methotrexate.

### 3.3. Quantitative Phase Imaging

After treatment, the cells were followed for 72 h with a Livecyte^TM^ kinetic cytometer (Phase Focus Ltd., Sheffield, UK). Livecyte^TM^, through ptychographic quantitative phase imaging (QPI), identifies individual cells and monitors their morphological and behavioral features in a label-free manner. All the experiments were performed in four replicates [28].

### 3.4. MTT Viability Assay

Cellular metabolic activity, as an indicator of cell viability and cytotoxicity, was measured by MTT assay in OS cells after 48 h and 72 h treatments. Briefly, 10µL of 3-(4,5-Dimethyl-2-thiazolyl)-2,5-diphenyl-2H-tetrazolium bromide (MTT) (5 mg/mL stock solution) (Merk KGaA, Darmstadt, Germany) were added to each well, and the plate was incubated for 2 h (5% CO_2_/humidified air at 37 °C). After 2 h, the cell culture medium was removed, and the formazan crystals were solubilized by adding 150µL/well of propan-2-ol (product reference, 34863; Merk KGaA, Darmstadt, Germany). The absorbance at 570 nm was measured and recorded with a multimode plate reader EnSpire^®^ (PerkinElmer, Waltham, USA). The sample absorbance at 690 nm was used as a reference wavelength for correction. All the experiments were performed in four replicates.

To test the selectivity of the most active molecules against cancer cells, normal human fibroblasts WS1 were treated for comparison. Cells were seeded and, after adhesion, the medium was removed and replaced with fresh medium containing 5, 10, 20, 40, and 80 µM of the selected compounds. DMSO was used as a negative control. At 48 h of treatment, the cellular metabolic activity was measured by the MTT assay, as previously described. 

### 3.5. Dose–Response Relationship and IC_50_ Calculation

To build the dose–response curve, cells were seeded at 5 × 10^3^ cells/well in a 96-well plate (15.000 cell/cm^2^) and incubated 24 h. Next, cells were treated with 80, 40, 20, 10, 5, 2.5, 1.25, and 0.6125 µM of the selected compounds. Cellular metabolic activity was measured by MTT assay 48 h after treatment, as previously described. The half-maximal inhibitory concentration (IC_50_) was determined using the log (inhibitory) vs. response–variable slope (four parameters) function of GraphPad Prism software, version 6.0c (GraphPad Software, San Diego, CA, USA). All the experiments were performed in a replicate of twelve.

### 3.6. Cell Cycle Analysis

MG63 cells were seeded at 1 × 10^4^ cell/cm^2^ in 56 cm^2^ Petri dishes, incubated 24 h to allow cell adhesion, and treated with the selected compounds at their IC_50_ values. Doxorubicin and methotrexate were tested at 0.5 and 0.1 µM, respectively. After 24 h of treatment, cells were detached, counted, and stained according to the protocol reported by Erba et al. [29], with minor adjustments. Briefly, 1 × 10^6^ cells were pelleted and resuspended in trisodium citrate 0.1% *w*/*v* (Sigma-Aldrich Chemie GmbH, Schnelldorf, Germany), RNase 10 μg/mL (Sigma-Aldrich Chemie GmbH, Schnelldorf, Germany), IGEPAL^®^ CA-630 0.01% *v*/*v* (Sigma-Aldrich Chemie GmbH, Schnelldorf, Germany), and 50 μg/mL propidium iodide (Sigma-Aldrich Chemie GmbH, Schnelldorf, Germany). After 30 min of incubation at 37 °C in the dark, cells were analyzed through a BRYTE HS Flow Cytometer (Bio-Rad, Hercules, CA, USA) equipped with a Xe/Hg lamp tuned at 480 nm. PI fluorescence was collected with an emission band centered at 600 nm, on a linear scale. DNA distribution in the cell cycle was analyzed by the MODFIT software (Verity).

### 3.7. Nuclei and F-Actin Staining

MG63 cells were seeded at 15 × 10^3^ cell/cm^2^ on a glass coverslip in a 12 well-plate and incubated for 24 h. Then, cells were treated with the different compounds at their IC_50_ values. After 24 h, cells were washed twice with PBS and fixed for 15 min in a 4% (*w*/*v*) paraformaldehyde solution (Sigma-Aldrich Chemie GmbH, Schnelldorf, Germany) at room temperature. After three washes with PBS, cells were stained for 45 min at room temperature with FITC-phalloidin (Sigma-Aldrich Chemie GmbH, Schnelldorf, Germany) and Hoechst 33342 (Sigma-Aldrich Chemie GmbH, Schnelldorf, Germany). FITC-phalloidin and Hoechst 33342 were solubilized in PBS at a final concentration of 1µg/mL. Finally, sample were washed and mounted with Mowiol^®^ 4-88 (product reference, 81381; Sigma-Aldrich Chemie GmbH, Schnelldorf, Germany) containing DABCO as antifading. Images were acquired with a Nikon eclipse 90i fluorescence microscopy (Nikon Corporation, Tokyo, Japan).

### 3.8. Annexin V/Propidium Staining

MG63 cells were seeded at 1 × 10^4^ cell/cm^2^ in 56 cm^2^ Petri dishes, incubated 24 h to allow cell adhesion, and treated with the selected compounds at their IC_50_ values. Doxorubicin and methotrexate were tested at 0.5 and 0.1 µM, respectively. After 18 h of treatment, cells were harvested and analyzed to determine cell apoptosis in accordance with Annexin V-FITC/PI Apoptosis Detection Kit (Elabscience, Houston, TX, USA). The population of apoptotic cells was determined and analyzed using Guava easyCyte 5HT flow cytometer (Luminex Corporation, Austin, TX, USA), with an excitation wavelength at 488 nm and an emission at 525 nm (FITC fluorescence) and 600 nm (PI fluorescence) on a logarithmic scale.

### 3.9. Statistical Analysis

A two-way ANOVA corrected for multiple comparisons (Dunnett test) was performed with GraphPad Prism software (GraphPad Software, version 6.0c, San Diego, CA, USA) (www.graphpad.com). All treatments were analyzed against control (DMSO). Significance was graphically reported as follows: * *p* < 0.05, ** *p* < 0.01, and *** *p* < 0.001.

## 4. Discussion and Conclusions

The cytotoxic activities of a series of chalcones, designed based on compounds retrieved from a wide in-house library as related to the OS active Lic-A, were investigated on MG63 cells through different but complementary approaches. Seven molecules (**1e**, **1h**, **1i**, **1k**, **1o**, **1q,** and **1r**) were selected due to their significant antiproliferative effects and a favorable selectivity profile with respect to healthy cells. Cell cycle progression analyses showed that they induced a remarkable block in G2-M, earlier and higher than Lic-A. Furthermore, they were seen to behave as apoptotic inducers by means of annexin V and PI staining and cytofluorimetric analysis.

In a SAR perspective, this study seems to indicate for this series of compounds a preferred ortho/meta substitution pattern on ring B, which appeared to play a more important role with respect to the electronic properties of the substituent. Nevertheless, among the selected molecules, the insertion of electron-withdrawing groups seemed to increase the antiproliferative activity and favor the proapoptotic effect. Moreover, the cytotoxicity observed with the new compounds proved to be dose-dependent and persistent, the latter feature drawing the hypothesis of Michael adduct formation with the biological target.

From this in-depth evaluation, some compounds emerged for their promising biological profile. In particular, the nitro-substituted **1q** and **1r** proved to be the most cytotoxic derivatives, showing the lowest IC_50_ values (10.6 and 12.2 µM, respectively) comparable to Lic-A (10.4 µM). Although the introduction of a nitro group could often lead to unspecific toxicity and this functional group may be regarded as a possible toxicophore, it is noteworthy that both compounds exhibited selectivity for OS cells, showing only **1q** a mild reduction of normal cell viability at 20 µM. **1q** and **1r** also showed proapoptotic properties, outlined by cell cycle analysis, in which the arrest in G2-M observed at 24 h was still evident at 48 h. Additionally, a pre-G1 peak in the DNA profile and phosphatidylserine exposure were revealed, confirming a remarkable apoptosis induction.

A peculiar behavior was observed for compound **1e**, substituted with an ortho-methoxy group that is widely found in natural products endowed with anticancer activity. In detail, **1e** showed the highest IC_50_ (42.5 µM) in the cytotoxicity assay, but proved to be the most potent in inducing a G2-M cell cycle arrest both at 24 and 48 h. Nevertheless, the induction of apoptosis appeared less pronounced with respect to other derivatives. Considering the lack of toxicity detected on normal cells up to 80 µM, this compound could be a promising candidate to be evaluated as a cell synchronizer that is able to block tumor cells in the G2-M phase and to increase the cytotoxic effects of antitumor drugs.

In summary, through reprofiling of selected in-house molecules and subsequent SAR studies, potential hit compounds showing antiproliferative activity on OS cells were obtained. These hits could further go through an optimization campaign to obtain new drug candidates for OS treatment. Along with the biological results obtained with this set of chalcones, this study could turn the spotlight on the advantages of an integrated methodological approach, exploiting both classic and innovative techniques. The combination of QPI with conventional assays, such as flow cytometric analysis, could allow researchers to obtain an efficient flowchart to screen large families of molecules and select the most promising compounds.

## Figures and Tables

**Table 1:** Structure of studied compounds **1a**–**s**, cell growth inhibition data, and IC_50_ values of selected compounds.

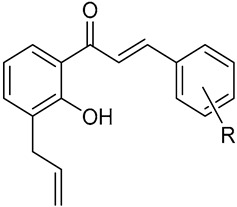			
Cpd	R	Cell Number (%) ^a^	IC_50_ (95% CI) ^b^ µM
**Lic-A**		**43.01 ± 9%**	**10.4 (9.4–11.4)**
1a ^c^	H	75.05 ± 4.58%	n.d.
1b ^c^	2-CH_3_	78.07 ± 5.72%	n.d.
1c ^c^	3-CH_3_	62.94 ± 4.55%	n.d.
1d ^c^	4-CH_3_	79.44 ± 11.12%	n.d.
**1e** ^c^	**2-OCH_3_**	**53.55 ± 6.87%**	**42.5 (22.9–78.8)**
1f ^c^	3-OCH_3_	64.51 ± 26.9%	n.d.
1g	4-OCH_3_	72.12 ± 9.37%	n.d.
**1h** ^c^	**2-Cl**	**54.26 ± 8.62%**	**17.0 (15.4–18.9)**
**1i**	**3-Cl**	**51.11 ± 3.66%**	**12.7 (11.5–14.0)**
1j ^c^	4-Cl	70.15 ± 6.85%	n.d.
**1k**	**2-OH**	**51.99 ± 3.33%**	**14.5 (12.0–17.5)**
1l	3-OH	64.26 ± 8.602%	n.d.
1m	4-OH	69.83 ± 6.35%	n.d.
1n	2-F	63.47 ± 9.66%	n.d.
**1o**	**3-F**	**52.55 ± 8.25%**	**13.8 (12.4–15.3)**
1p	4-F	71.67 ± 9.72%	n.d.
**1q**	**2-NO_2_**	**45.18 ± 10.94%**	**10.6 (10.2–11.1)**
**1r**	**3-NO_2_**	**45.66 ± 4.61%**	**12.2 (11.6–12.8)**
1s	4-NO_2_	84.16 ± 12.46%	n.d.

^a^ Cell number is reported as percentage with respect to control after 48 h treatment with 10 µM of each compound; ^b^ Confidence Interval; ^c^ Ref. [19]; and n.d. not determined.

## Data Availability

Not applicable.

## Supplementary Materials

The following supporting information can be downloaded at: https://www.mdpi.com/article/10.3390/molecules27123751/s1, Figure S1: Cell cycle analysis; Figure S2: Effects of Lic-A analogs on cell morphology in MG63 cells; 1H- and 13C-NMR spectra of compounds **1g**, **1i**, and **1k**–**s**.

## Sample Availability

Samples of the compounds are available from the authors.
