# Supplementary material for: Natural-like Chalcones with Antitumor Activity on Human MG63 Osteosarcoma Cells
Source: Molecules. 2022 Jun 11;27(12):3751. doi: 10.3390/molecules27123751 (PMC9229256; doi:10.3390/molecules27123751)
Supplement: Supplementary file 1 [file molecules-27-03751-s001.zip › molecules-1723659-supplementary.pdf]

Supplementary Material

Natural-like chalcones with antitumor activity on human MG63 osteosarcoma cells

Martina Rossi,[1] Concettina Cappadone,[1] Giovanna Picone,[1] Alessandra Bisi,[2] Giovanna Farruggia,[1] Federica Belluti,[2] Paolo Blasi,[1] Silvia Gobbi,[2] Emil Malucelli[1]

Department of Pharmacy and Biotechnology, Alma Mater Studiorum-University of Bologna [1]Via San Donato 19/2, 40127, Bologna, Italy [2]Via Belmeloro 6, 40126, Bologna, Italy

Contents

Figures S1 and S2 S2

^{1}H- and ^{13}C-NMR spectra of compounds 1g, 1i, 1k-s S3

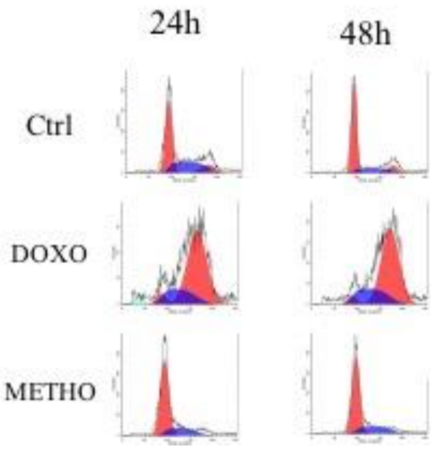

Figure S1. Cell cycle analysis. Cell cycle analysis after 24 or 48 h of treatment with doxorubicin and methotrexate at 0.5 and 0.1 μM respectively. Histograms show a representative experiment of the distribution of cell cycle phase.

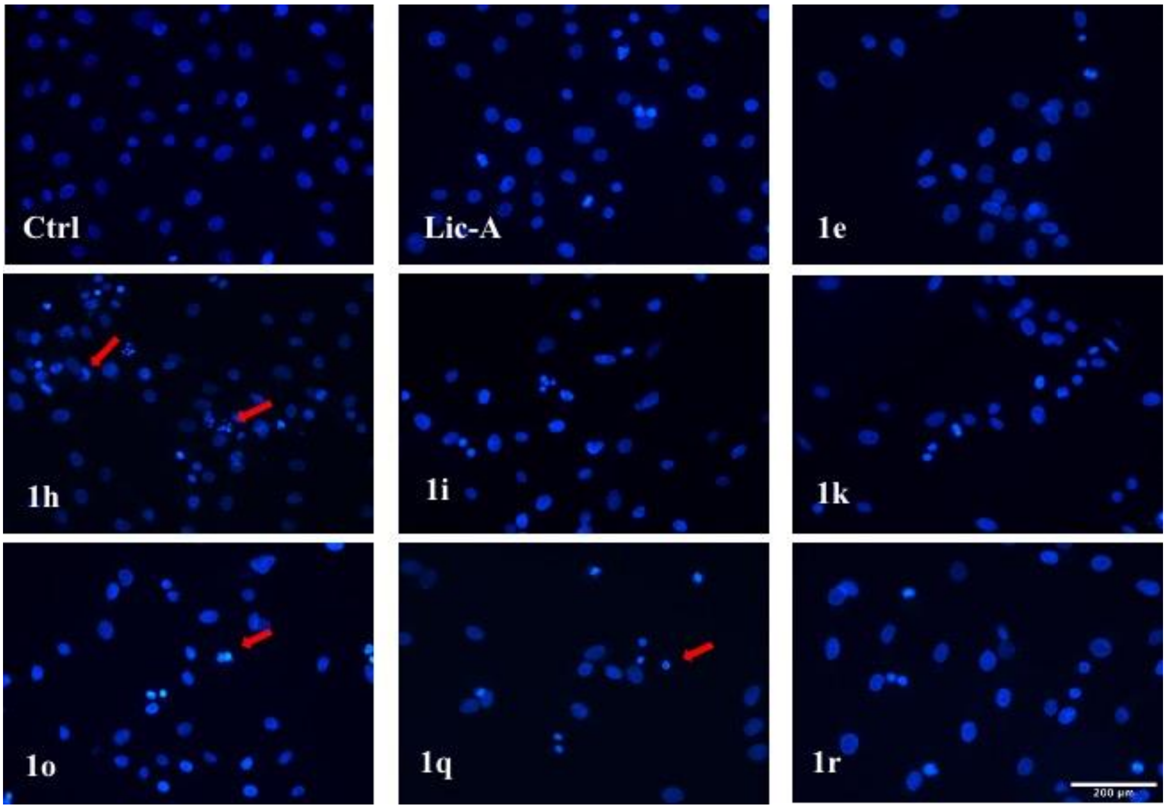

Figure S2. Effects of Lic-A analogs on cell morphology in MG63 cells. Fluorescence microscopy of control and treated cells with IC_{50} dose for 24 h. Nuclei are stained in blue with Hoechst. The arrows indicated the fragmented and pycnotic nuclei and condensed chromatin. Scale bar 200 μm.

^{1}H- and ^{13}C-NMR spectra of compounds 1g, 1i, 1k-s

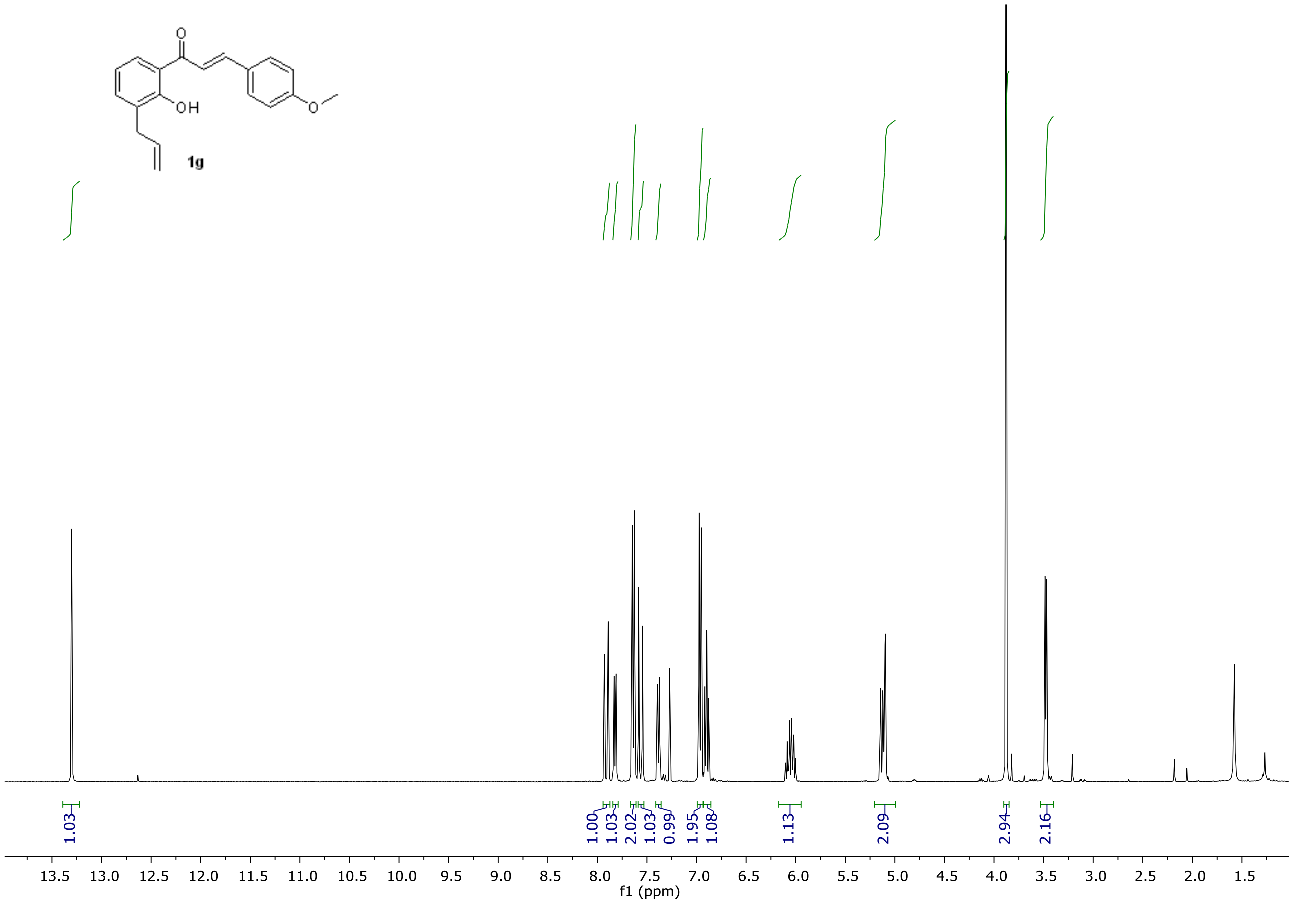

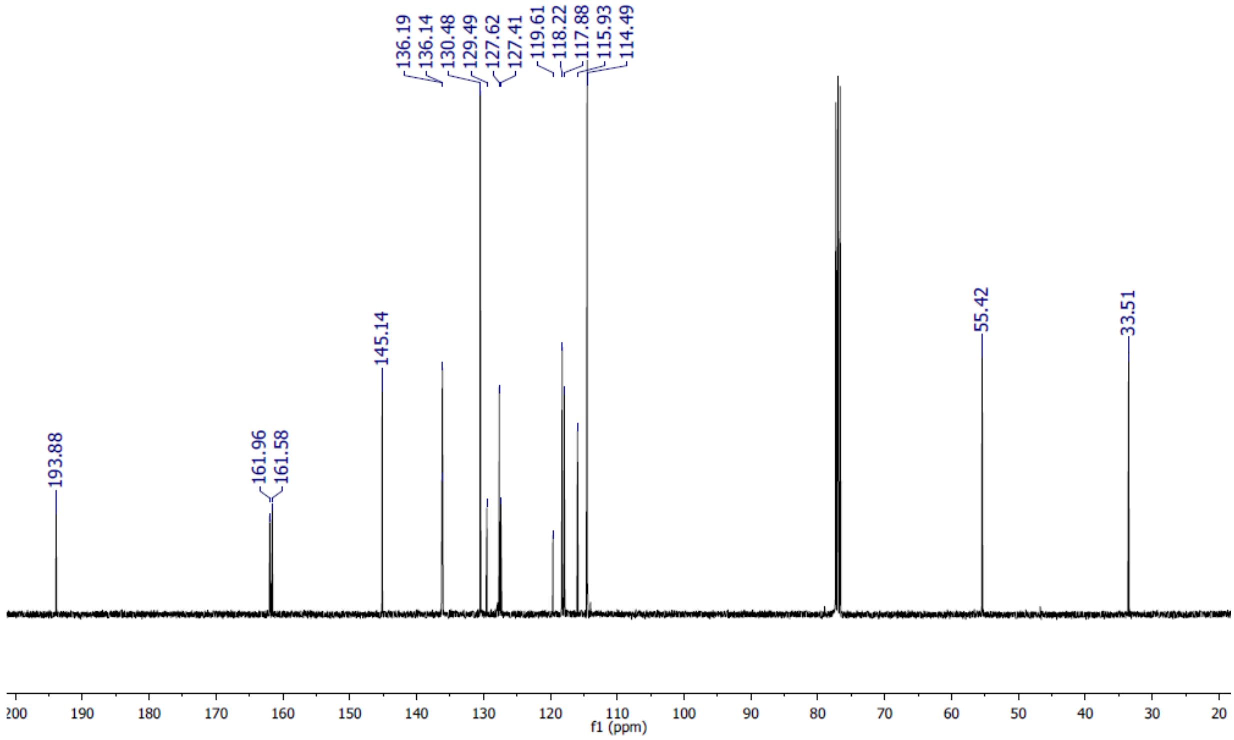

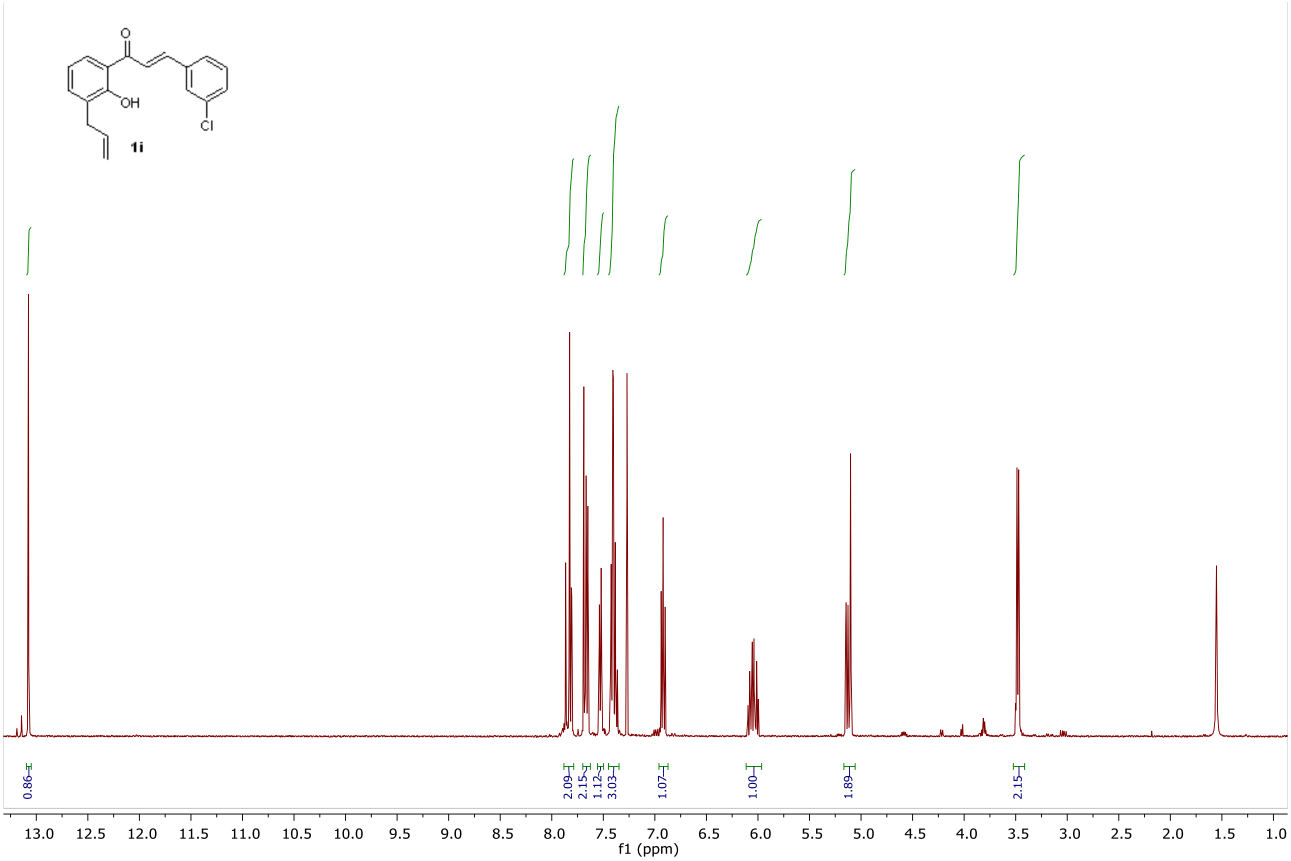
O
OH
Cl
1i
0.86
2.09
2.15
1.12
3.03
1.07
1.00
1.89
2.15
13.0 12.5 12.0 11.5 11.0 10.5 10.0 9.5 9.0 8.5 8.0 7.5 7.0 6.5 6.0 5.5 5.0 4.5 4.0 3.5 3.0 2.5 2.0 1.5 1.0
f1 (ppm)

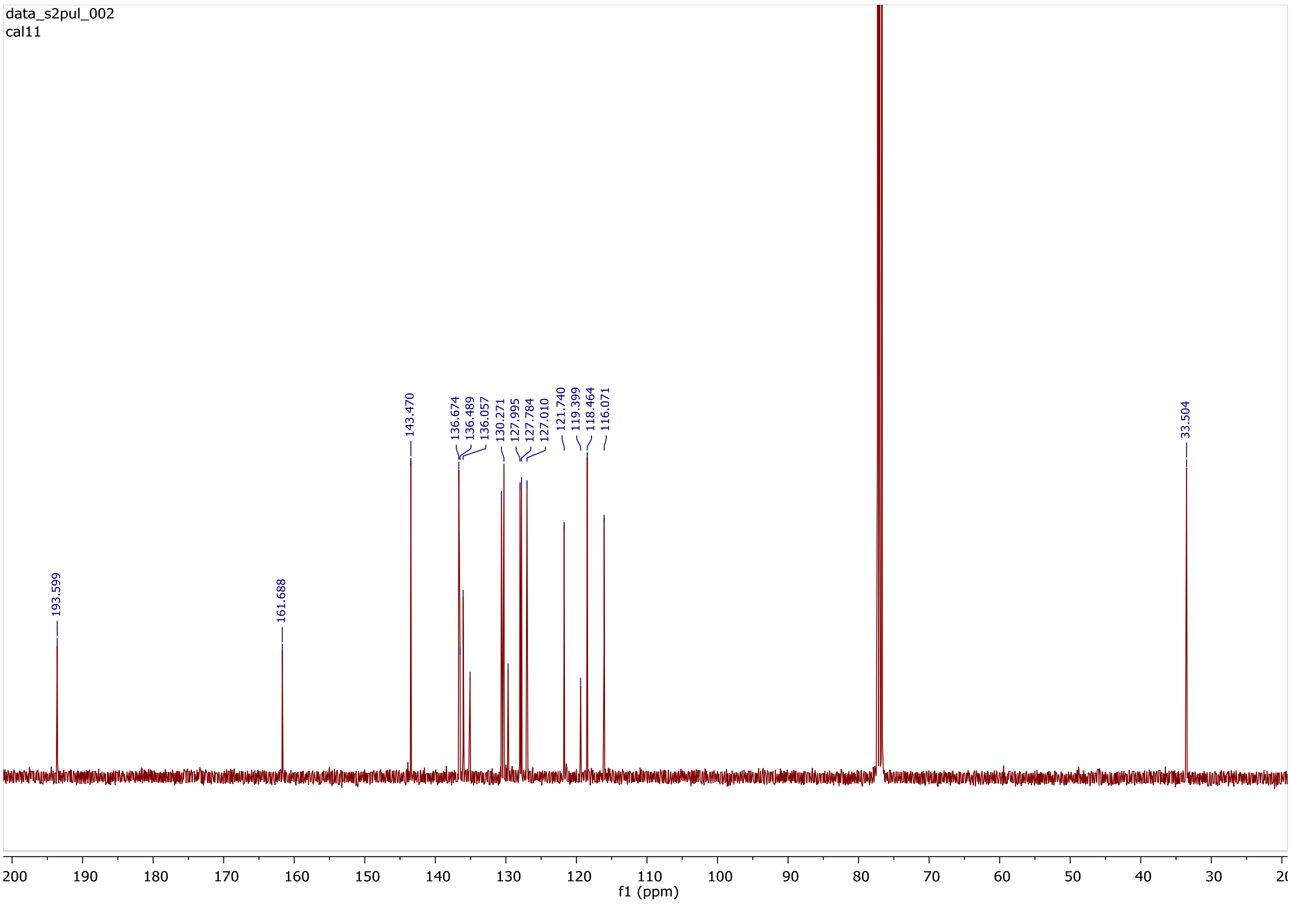
data_s2pul_002
cal11
193.599
161.688
143.470
136.674
136.489
136.057
130.271
127.995
127.784
127.010
121.740
119.399
118.464
116.071
33.504
200 190 180 170 160 150 140 130 120 110 100 90 80 70 60 50 40 30
f1 (ppm)

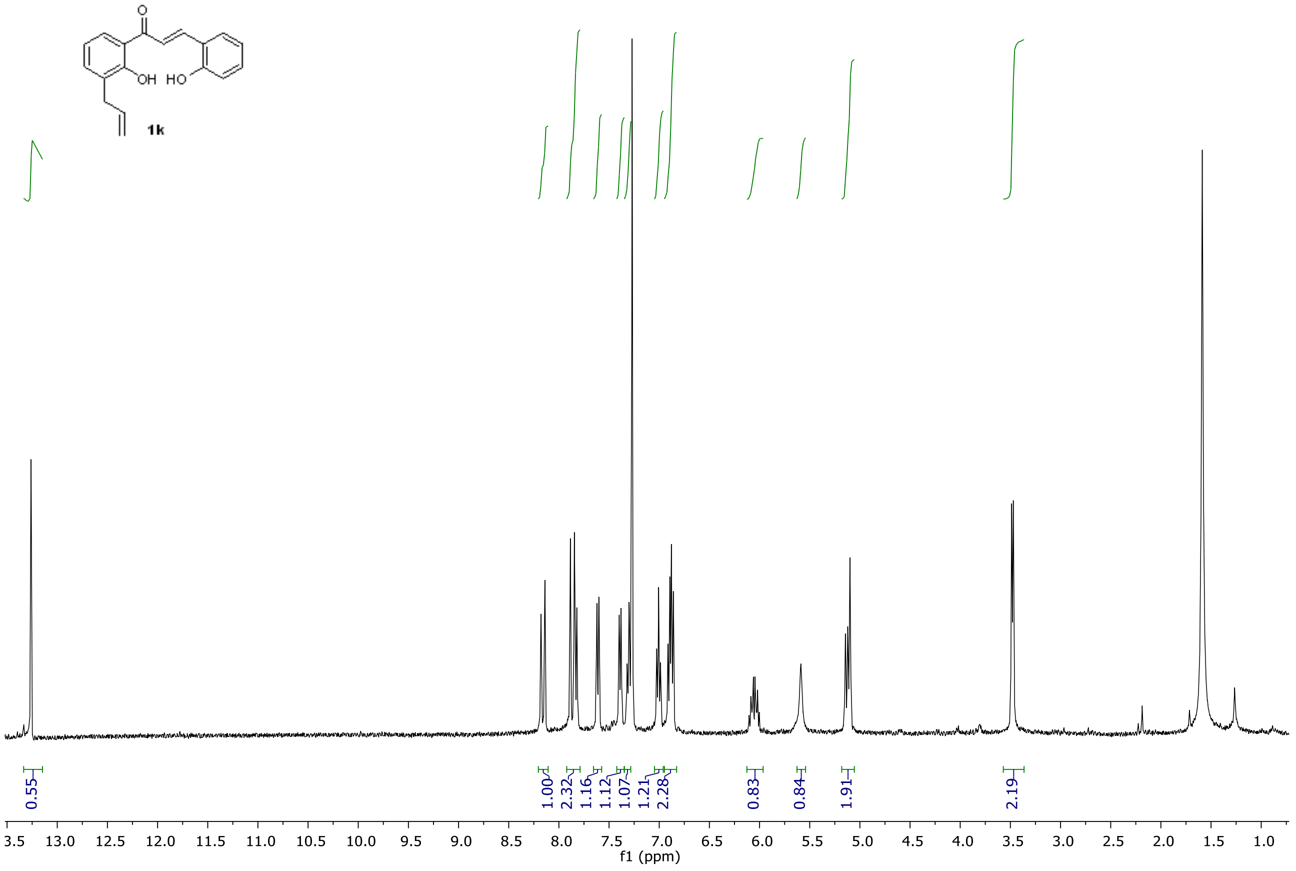

OH HO
1k
0.55
1.00
2.32
1.16
1.12
1.07
1.21
2.28
0.83
0.84
1.91
2.19
f1 (ppm)

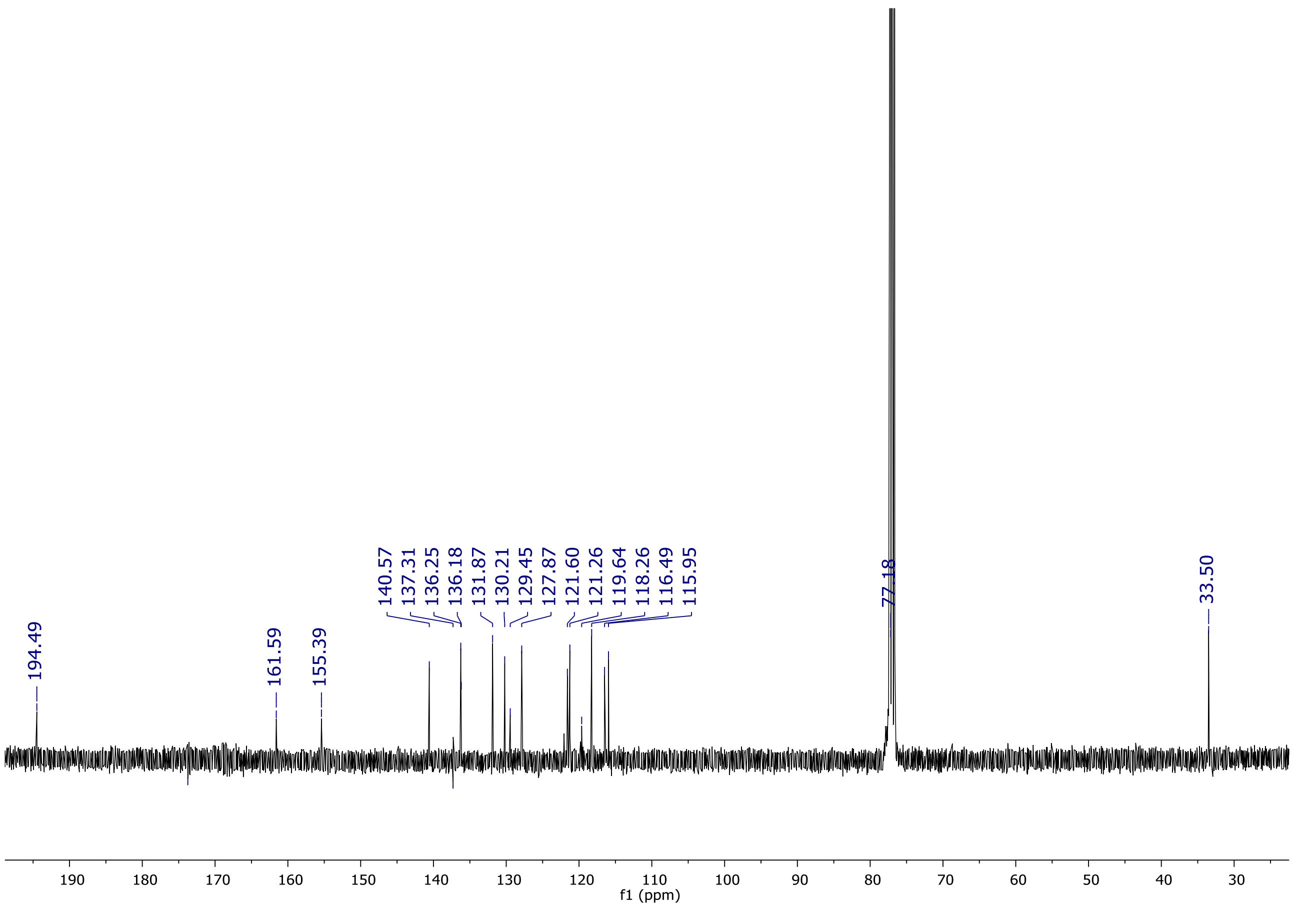

194.49
161.59
155.39
140.57
137.31
136.25
136.18
131.87
130.21
129.45
127.87
121.60
121.26
119.64
118.26
116.49
115.95
77.18
33.50
f1 (ppm)

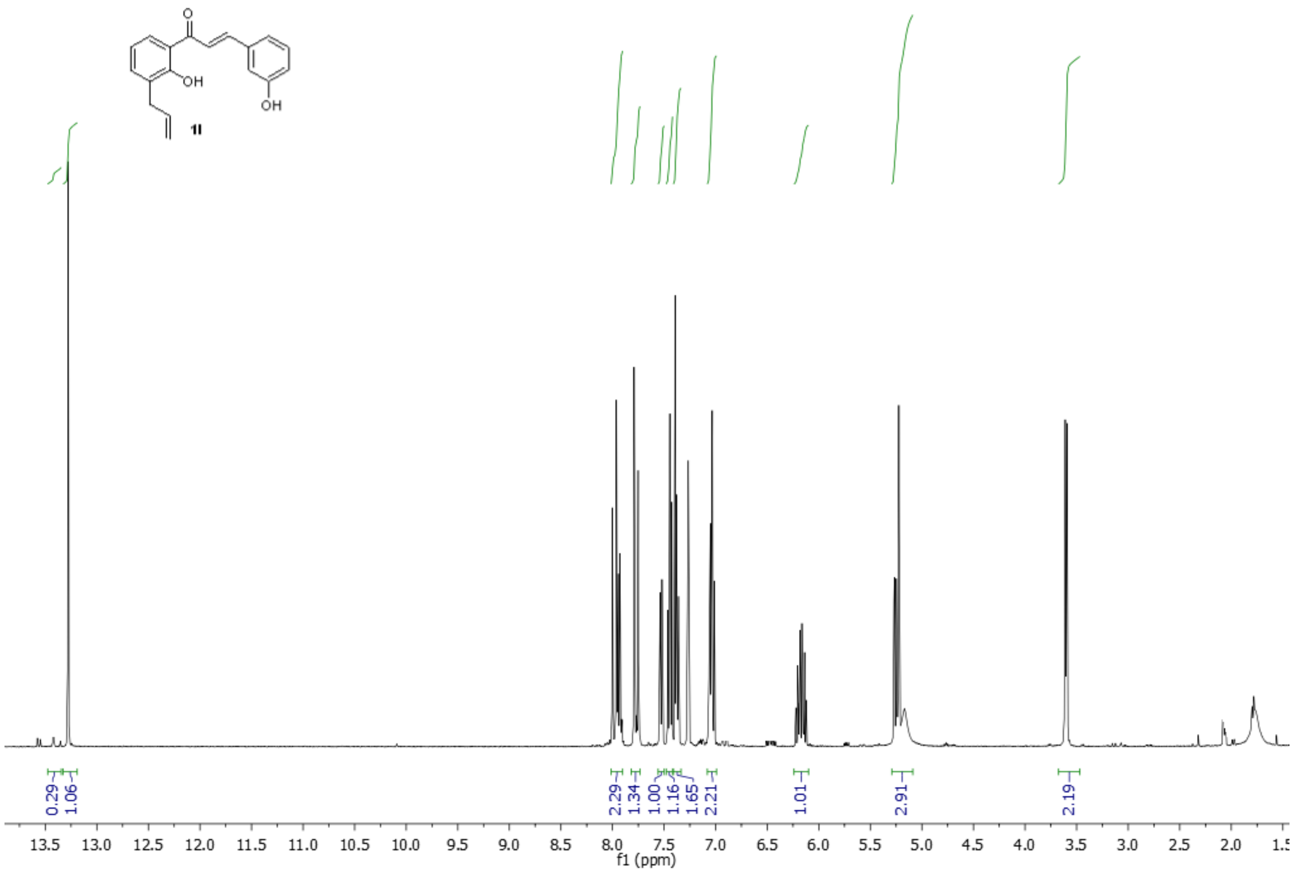
O
OH
OH
1l
0.29
1.06
2.29
1.34
1.00
1.16
1.65
2.21
1.01
2.91
2.19
13.5 13.0 12.5 12.0 11.5 11.0 10.5 10.0 9.5 9.0 8.5 8.0 7.5 7.0 6.5 6.0 5.5 5.0 4.5 4.0 3.5 3.0 2.5 2.0
f1 (ppm)

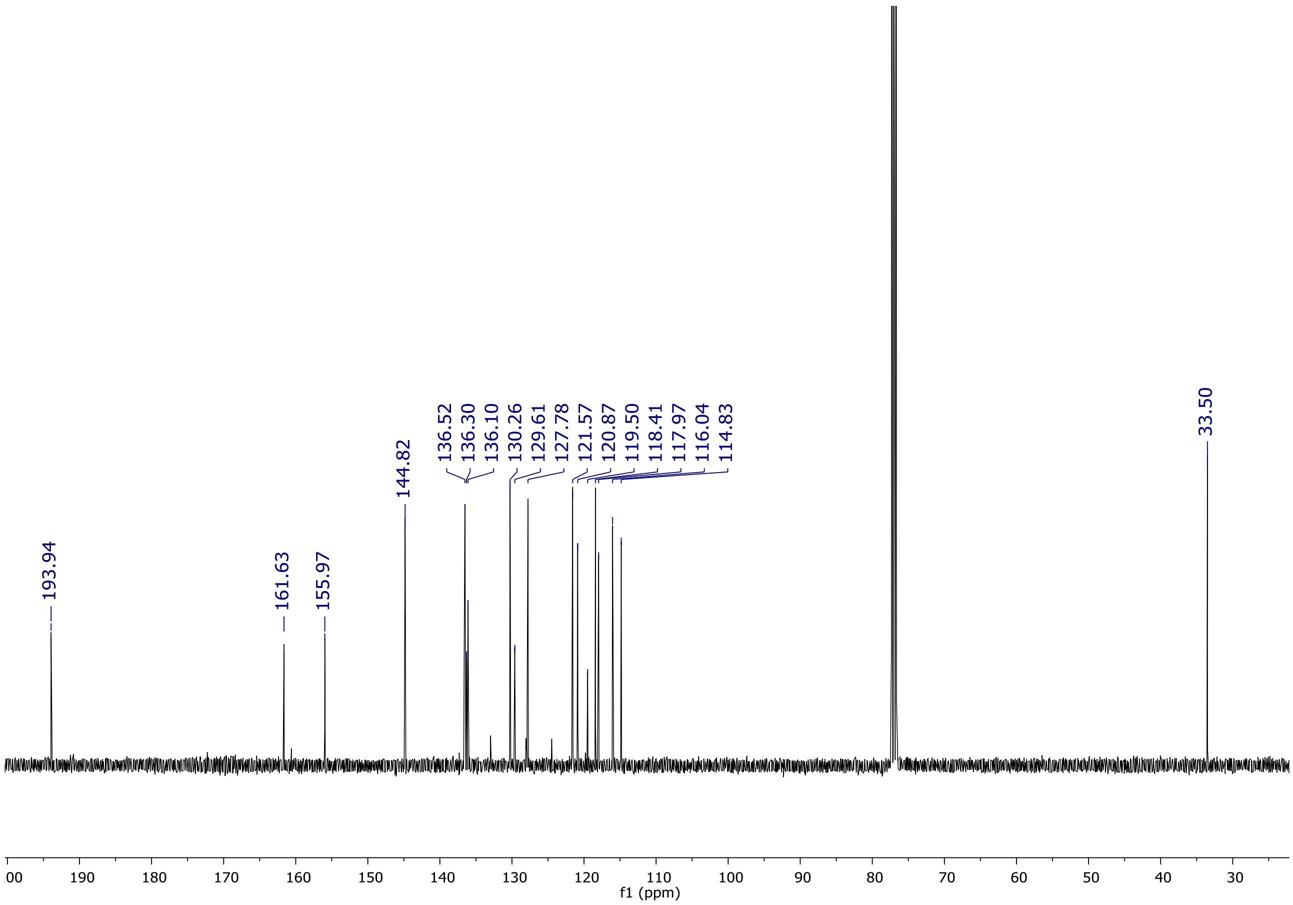
193.94
161.63
155.97
144.82
136.52
136.30
136.10
130.26
129.61
127.78
121.57
120.87
119.50
118.41
117.97
116.04
114.83
33.50
190 180 170 160 150 140 130 120 110 100 90 80 70 60 50 40 30
f1 (ppm)

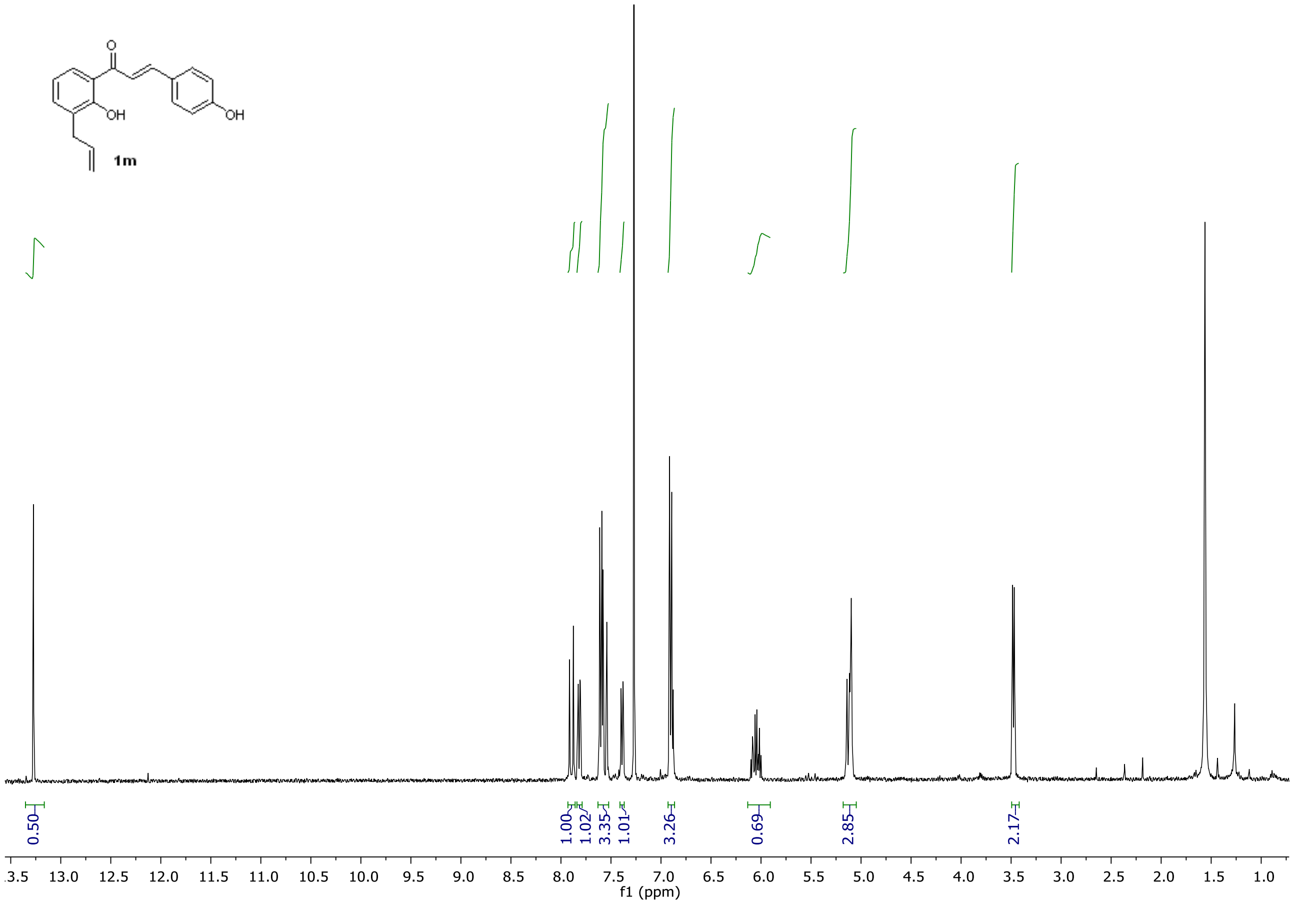
O
OH
OH
1m
0.50
1.00
1.02
3.35
1.01
3.26
0.69
2.85
2.17
3.5 13.0 12.5 12.0 11.5 11.0 10.5 10.0 9.5 9.0 8.5 8.0 7.5 7.0 6.5 6.0 5.5 5.0 4.5 4.0 3.5 3.0 2.5 2.0 1.5 1.0
f1 (ppm)

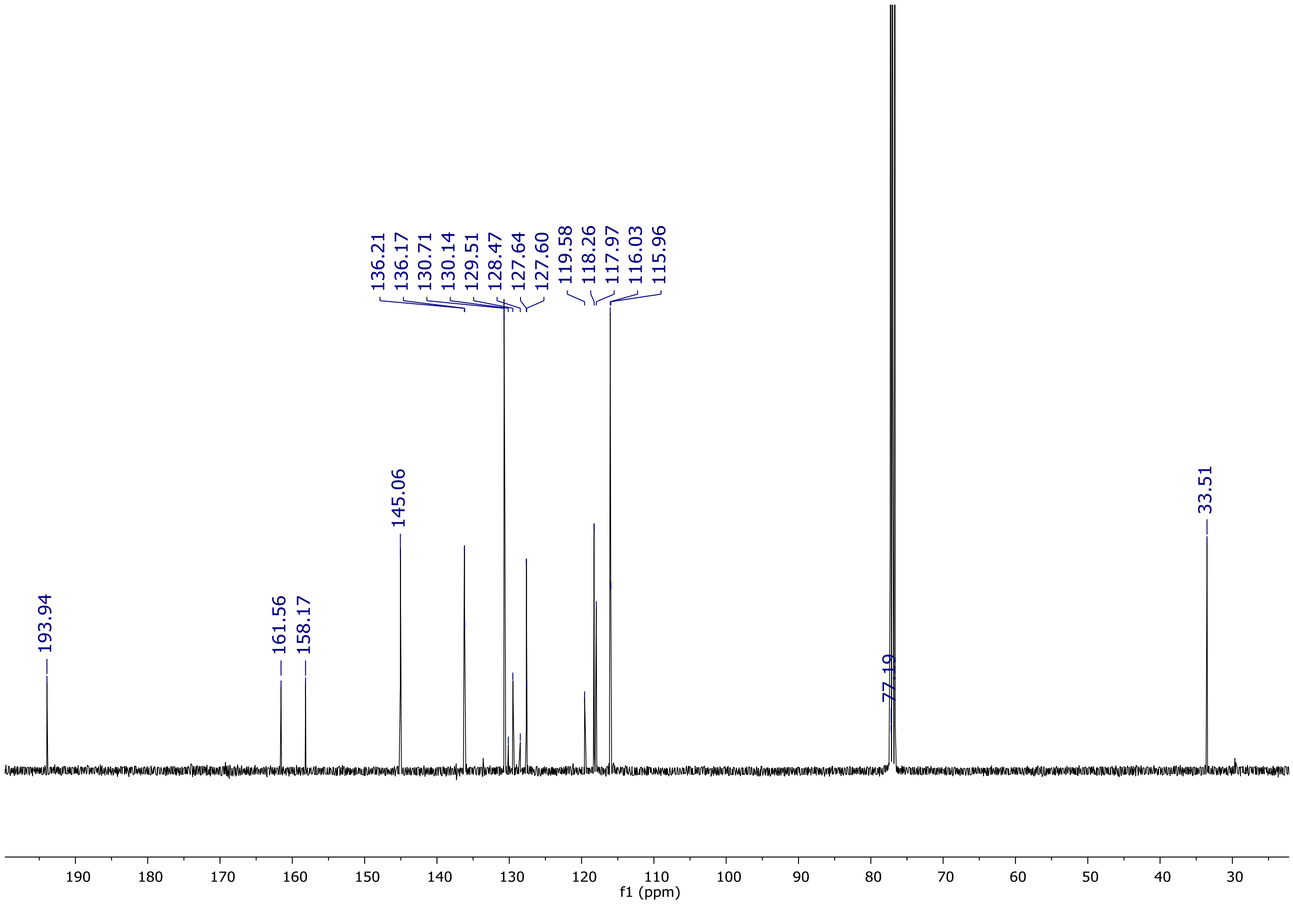
136.21
136.17
130.71
130.14
129.51
128.47
127.64
127.60
119.58
118.26
117.97
116.03
115.96
193.94
161.56
158.17
145.06
77.19
33.51
190 180 170 160 150 140 130 120 110 100 90 80 70 60 50 40 30
f1 (ppm)

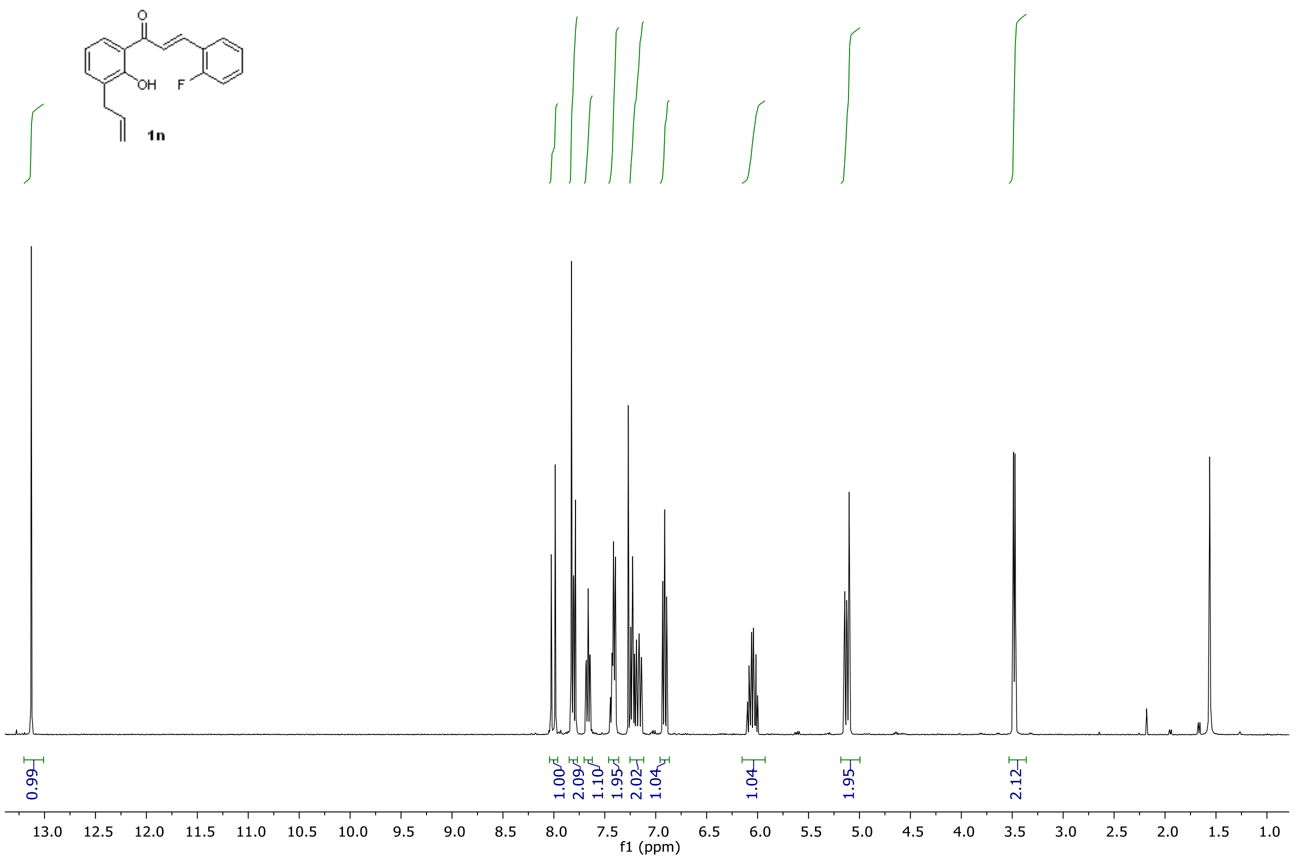

OH
F
O
1n
0.99
1.00
2.09
1.10
1.95
2.02
1.04
1.04
1.95
2.12
13.0 12.5 12.0 11.5 11.0 10.5 10.0 9.5 9.0 8.5 8.0 7.5 7.0 6.5 6.0 5.5 5.0 4.5 4.0 3.5 3.0 2.5 2.0 1.5 1.0
f1 (ppm)

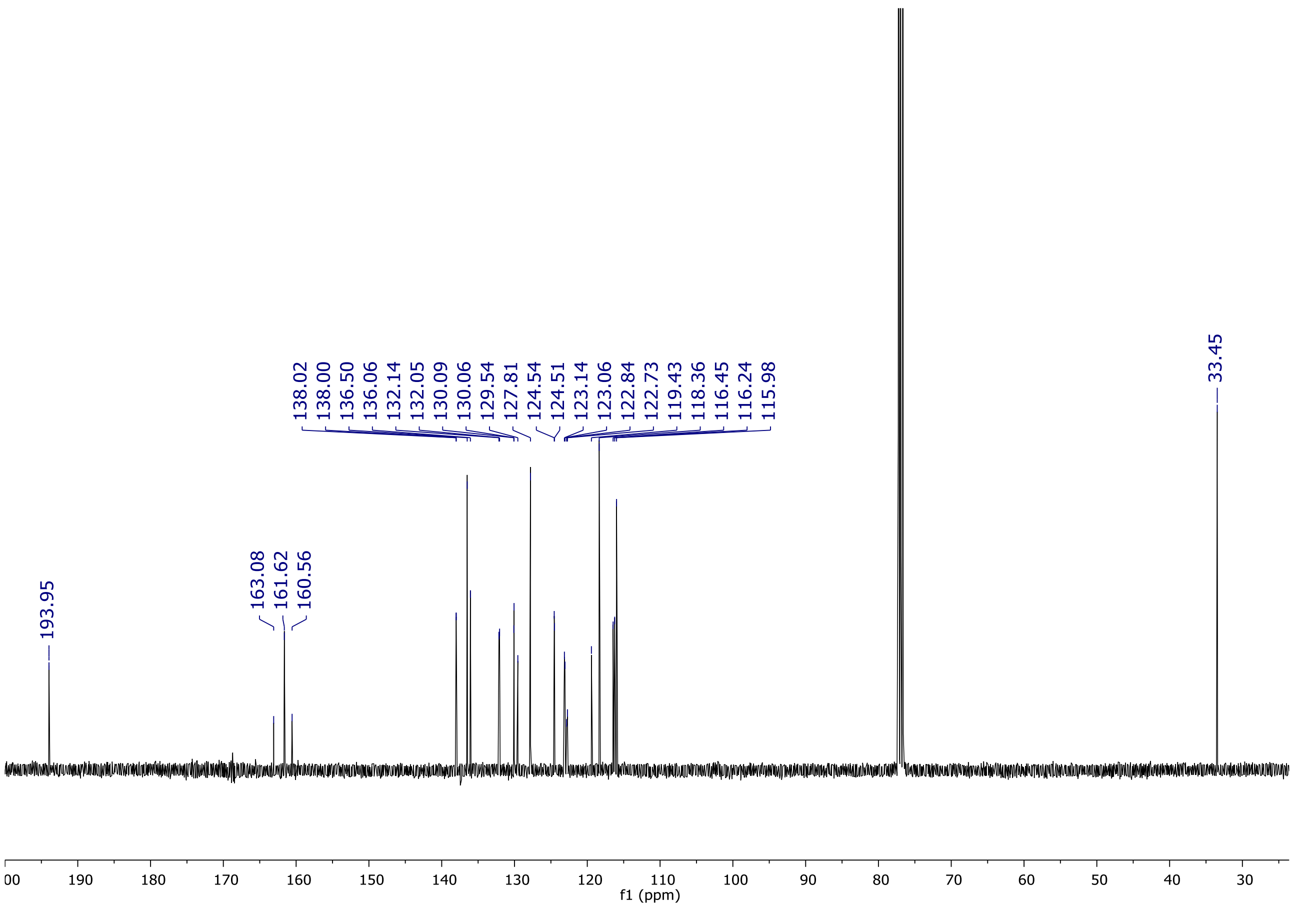

193.95
163.08
161.62
160.56
138.02
138.00
136.50
136.06
132.14
132.05
130.09
130.06
129.54
127.81
124.54
124.51
123.14
123.06
122.84
122.73
119.43
118.36
116.45
116.24
115.98
33.45
00 190 180 170 160 150 140 130 120 110 100 90 80 70 60 50 40 30
f1 (ppm)

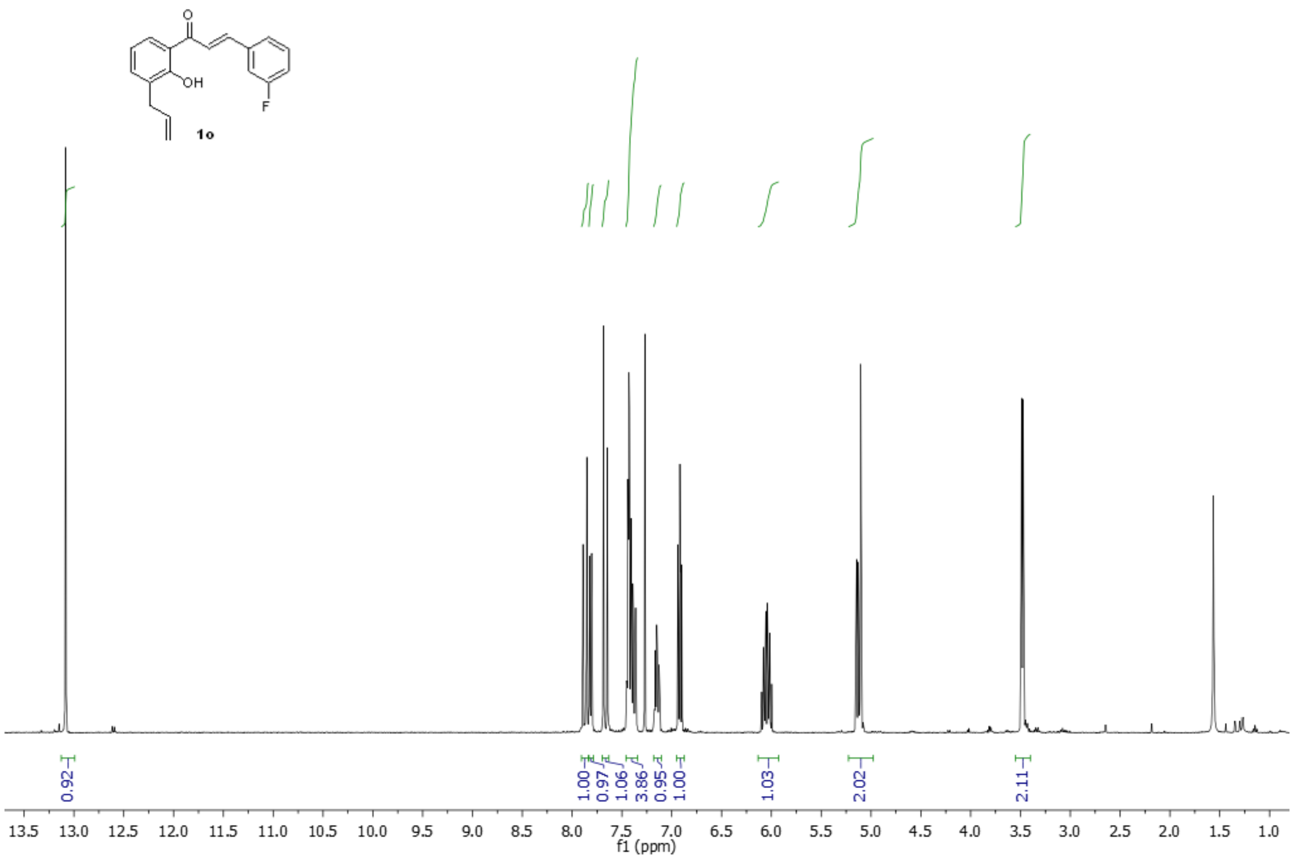

1o
0.92
1.00
0.97
1.06
3.86
0.95
1.00
1.03
2.02
2.11
13.5 13.0 12.5 12.0 11.5 11.0 10.5 10.0 9.5 9.0 8.5 8.0 7.5 7.0 6.5 6.0 5.5 5.0 4.5 4.0 3.5 3.0 2.5 2.0 1.5 1.0
f1 (ppm)

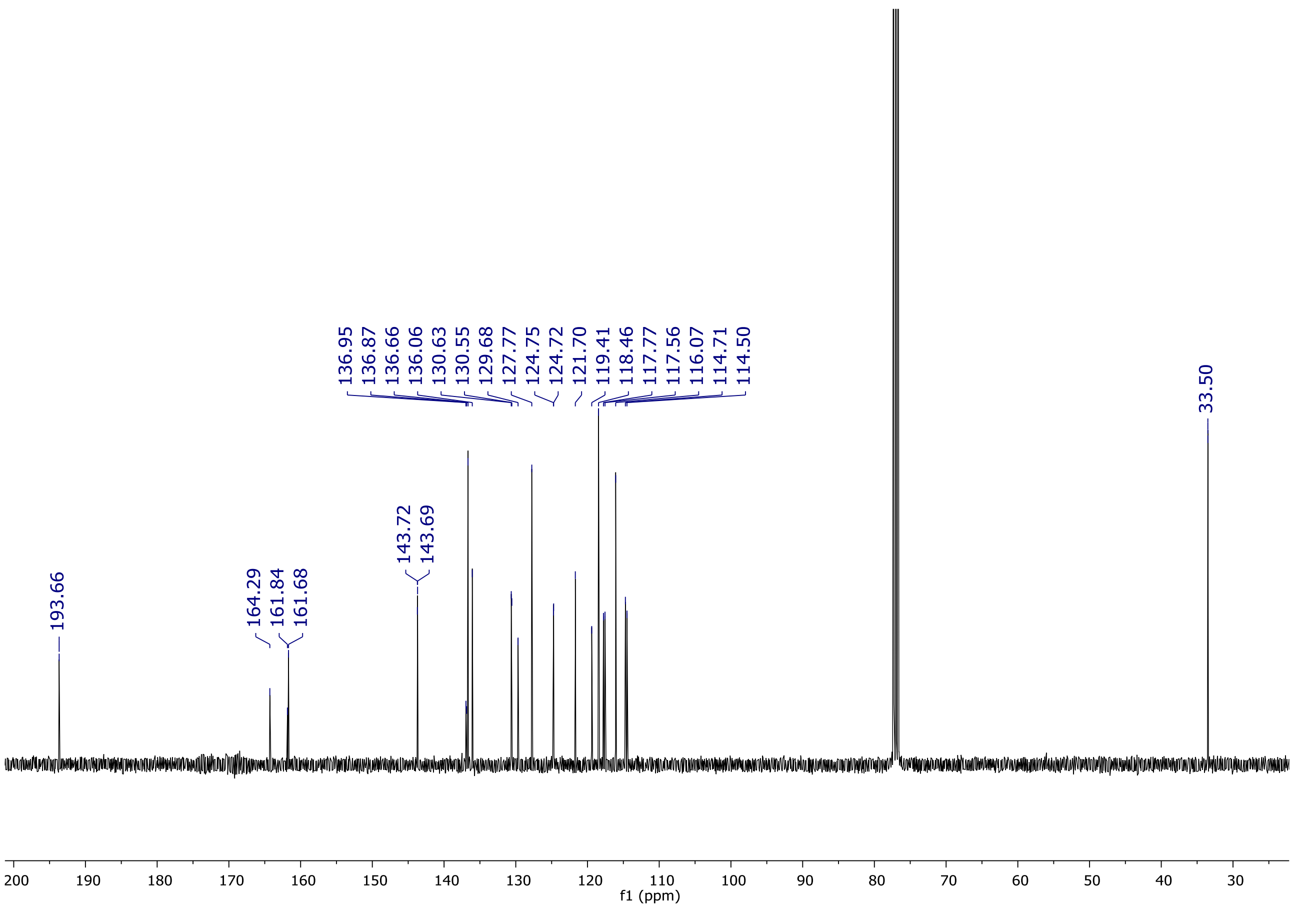

193.66
164.29
161.84
161.68
143.72
143.69
136.95
136.87
136.66
136.06
130.63
130.55
129.68
127.77
124.75
124.72
121.70
119.41
118.46
117.77
117.56
116.07
114.71
114.50
33.50
200 190 180 170 160 150 140 130 120 110 100 90 80 70 60 50 40 30
f1 (ppm)

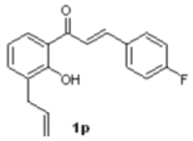
O
OH
F

1p

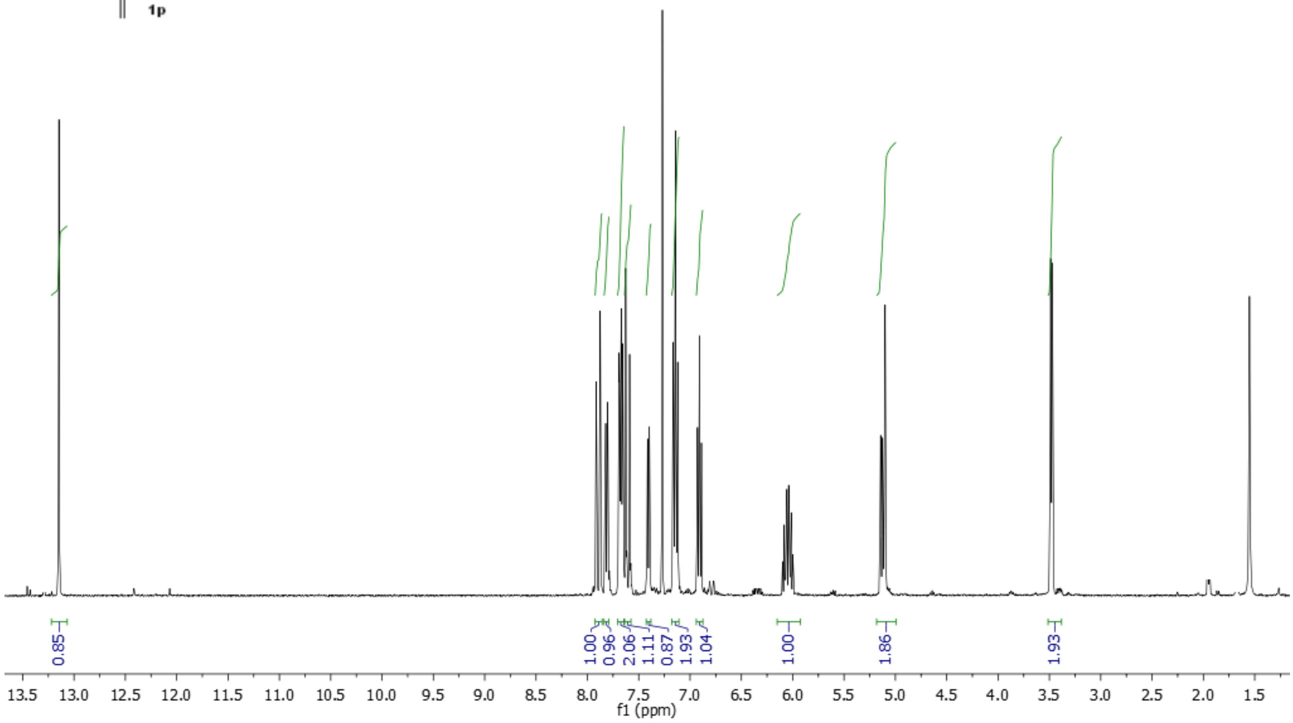
0.85
1.00
0.96
2.06
1.11
0.87
1.93
1.04
1.00
1.86
1.93
13.5 13.0 12.5 12.0 11.5 11.0 10.5 10.0 9.5 9.0 8.5 8.0 7.5 7.0 6.5 6.0 5.5 5.0 4.5 4.0 3.5 3.0 2.5 2.0 1.5
f1 (ppm)

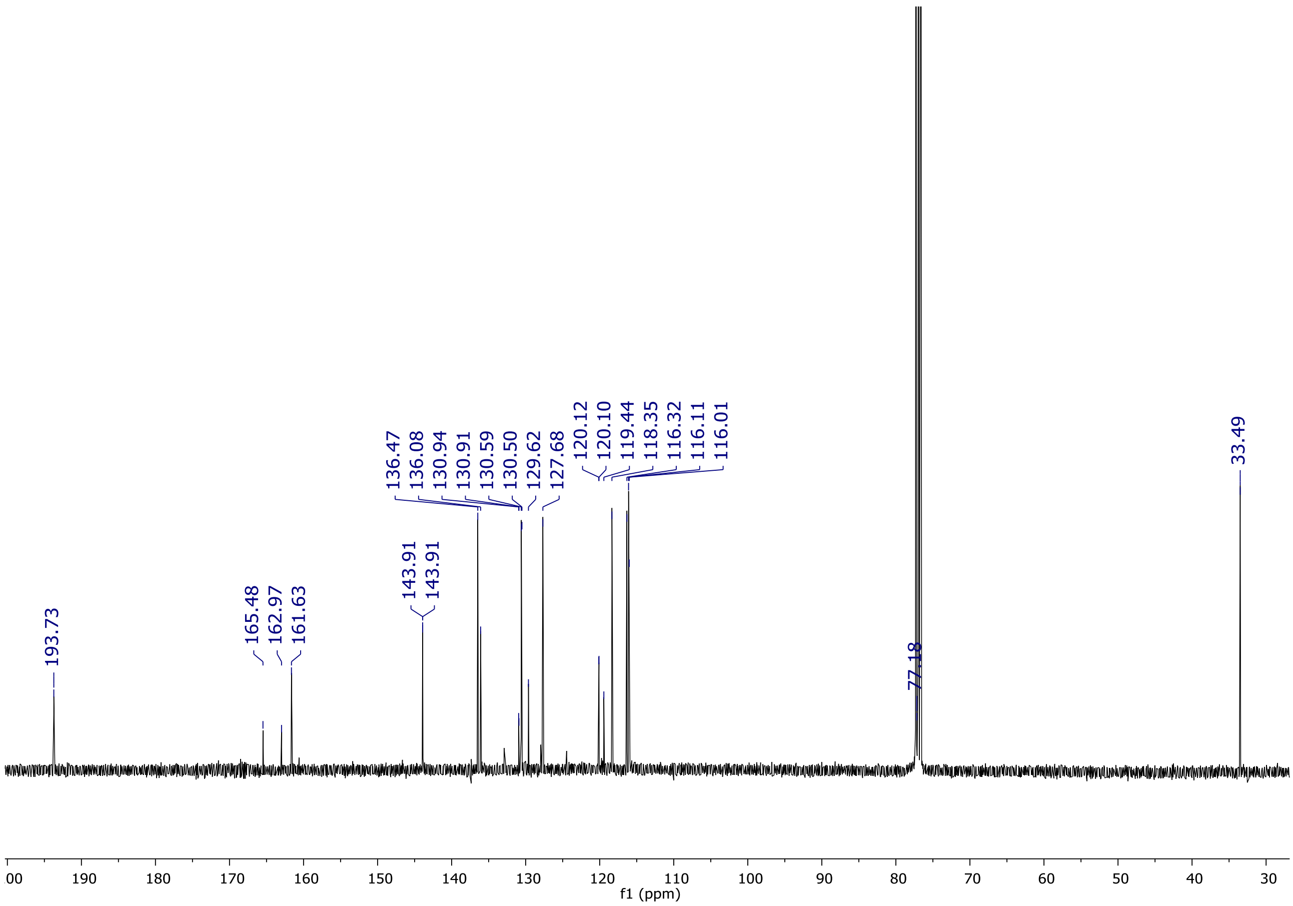
193.73
165.48
162.97
161.63
143.91
143.91
136.47
136.08
130.94
130.91
130.59
130.50
129.62
127.68
120.12
120.10
119.44
118.35
116.32
116.11
116.01
77.18
33.49
00 190 180 170 160 150 140 130 120 110 100 90 80 70 60 50 40 30
f1 (ppm)

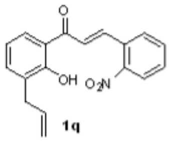
O
OH
O2N

1q

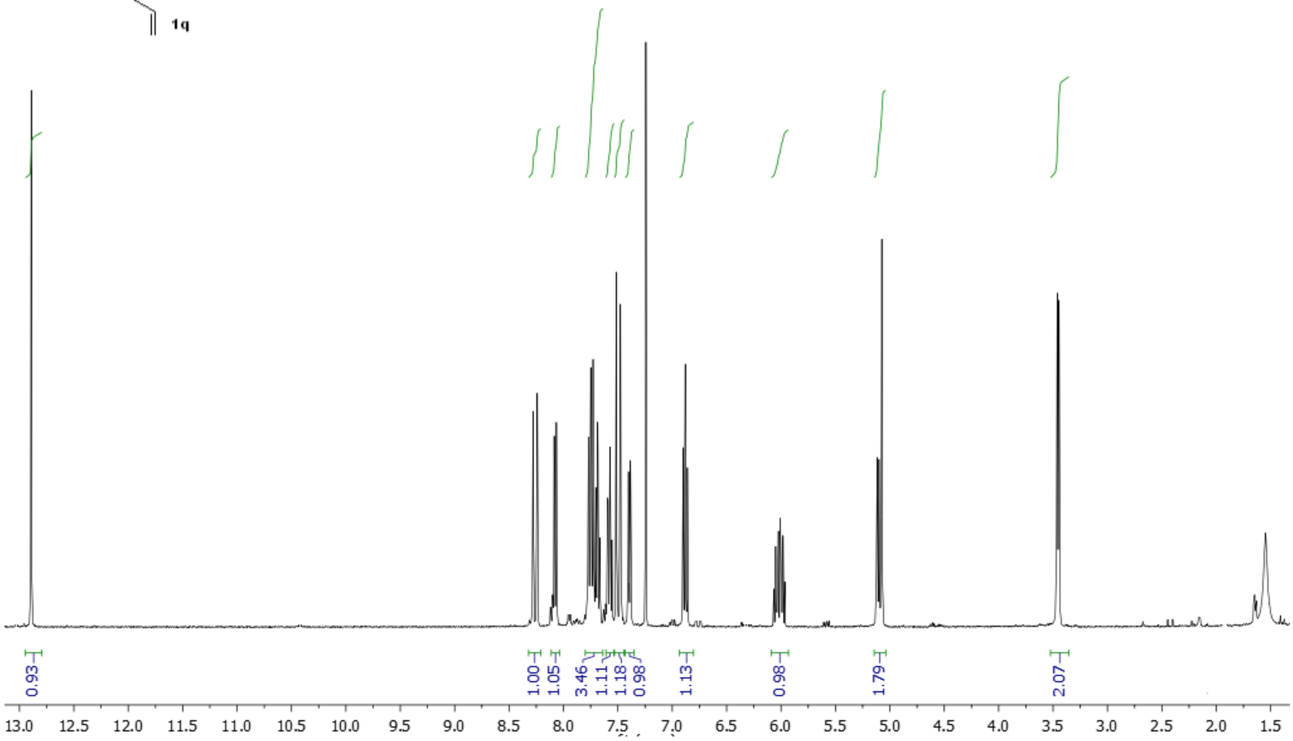
0.93
1.00
1.05
3.46
1.11
1.18
0.98
1.13
0.98
1.79
2.07
13.0 12.5 12.0 11.5 11.0 10.5 10.0 9.5 9.0 8.5 8.0 7.5 7.0 6.5 6.0 5.5 5.0 4.5 4.0 3.5 3.0 2.5 2.0 1.5

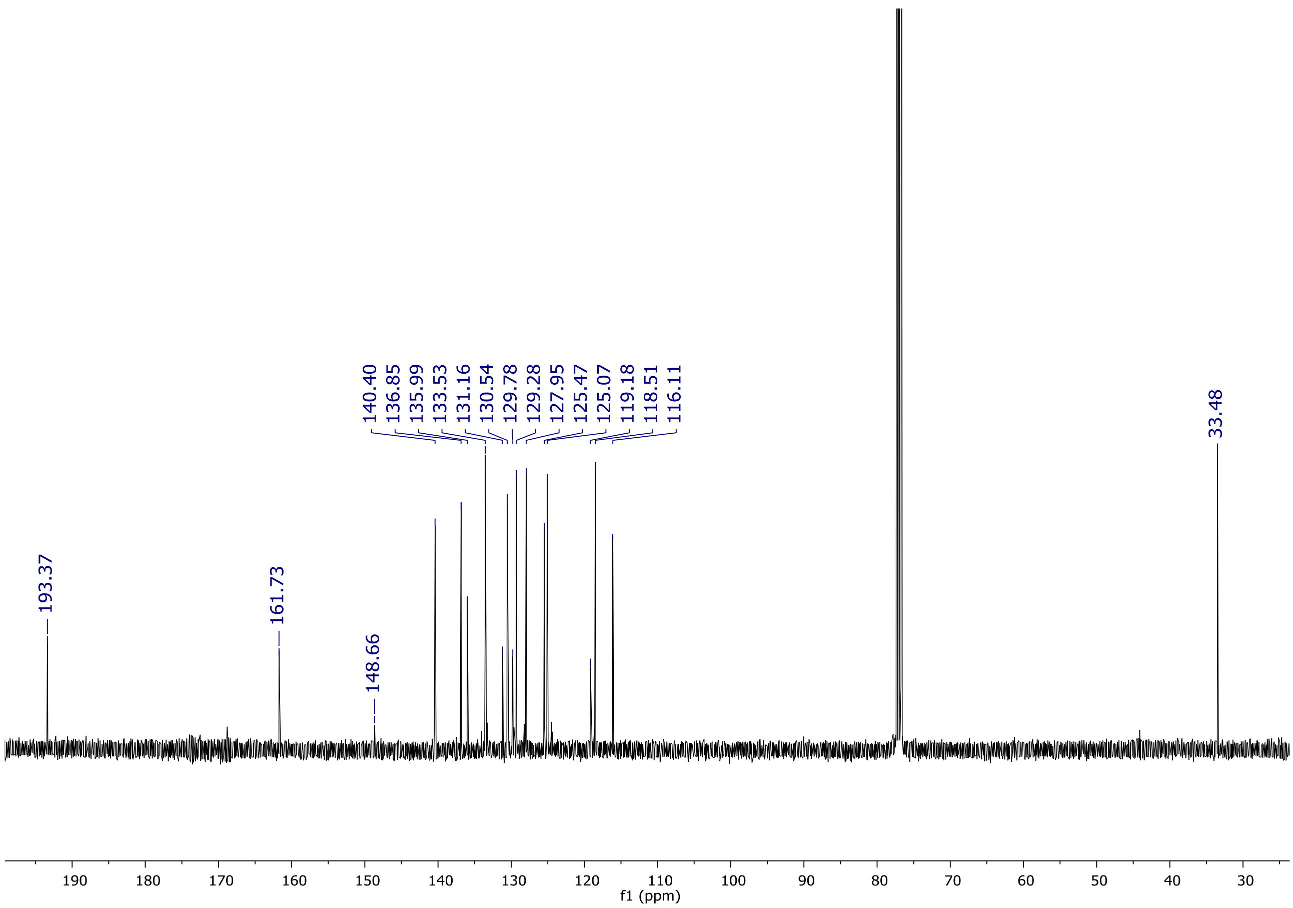
193.37
161.73
148.66
140.40
136.85
135.99
133.53
131.16
130.54
129.78
129.28
127.95
125.47
125.07
119.18
118.51
116.11
33.48
190 180 170 160 150 140 130 120 110 100 90 80 70 60 50 40 30
f1 (ppm)

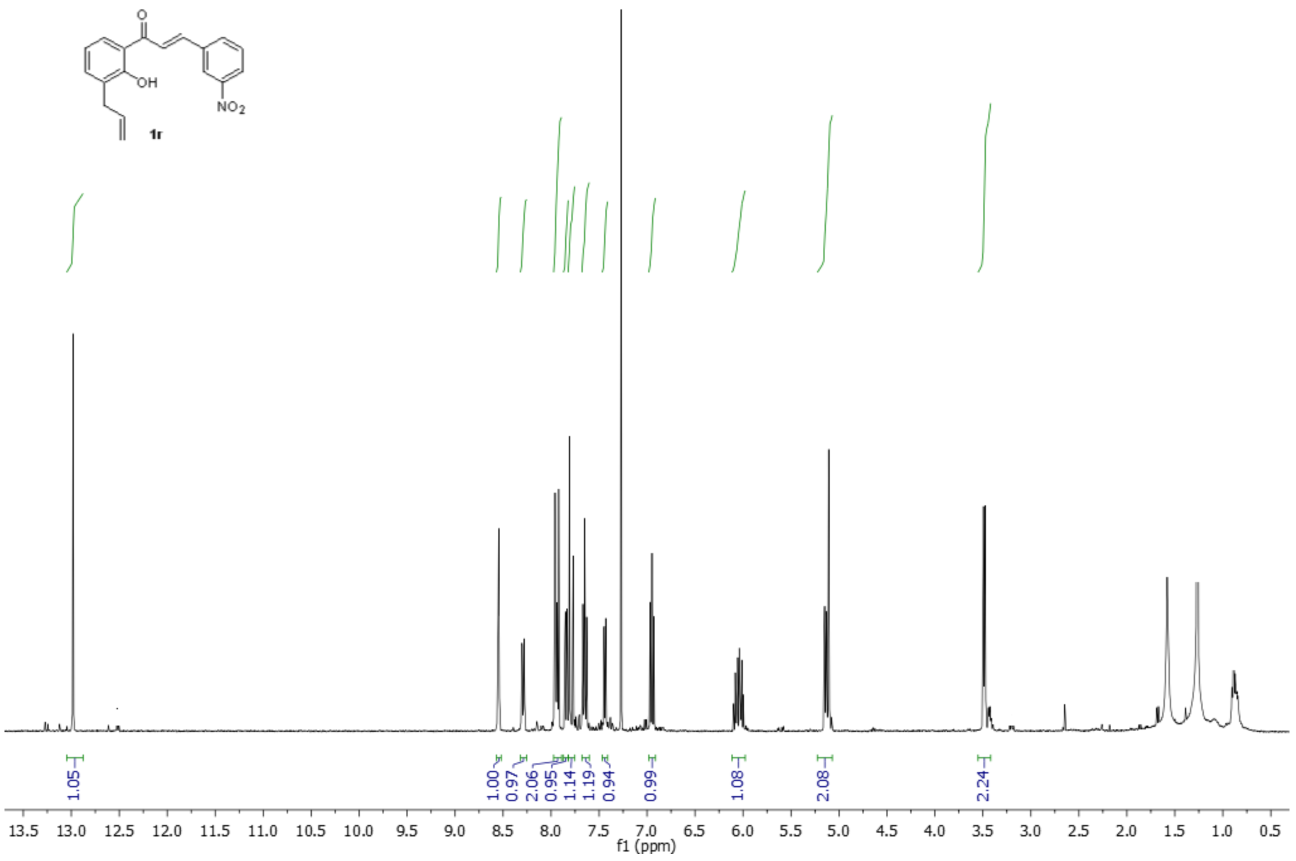
OH
NO2
1r
1.05
1.00
0.97
2.06
0.95
1.14
1.19
0.94
0.99
1.08
2.08
2.24
13.5 13.0 12.5 12.0 11.5 11.0 10.5 10.0 9.5 9.0 8.5 8.0 7.5 7.0 6.5 6.0 5.5 5.0 4.5 4.0 3.5 3.0 2.5 2.0 1.5 1.0 0.5
f1 (ppm)

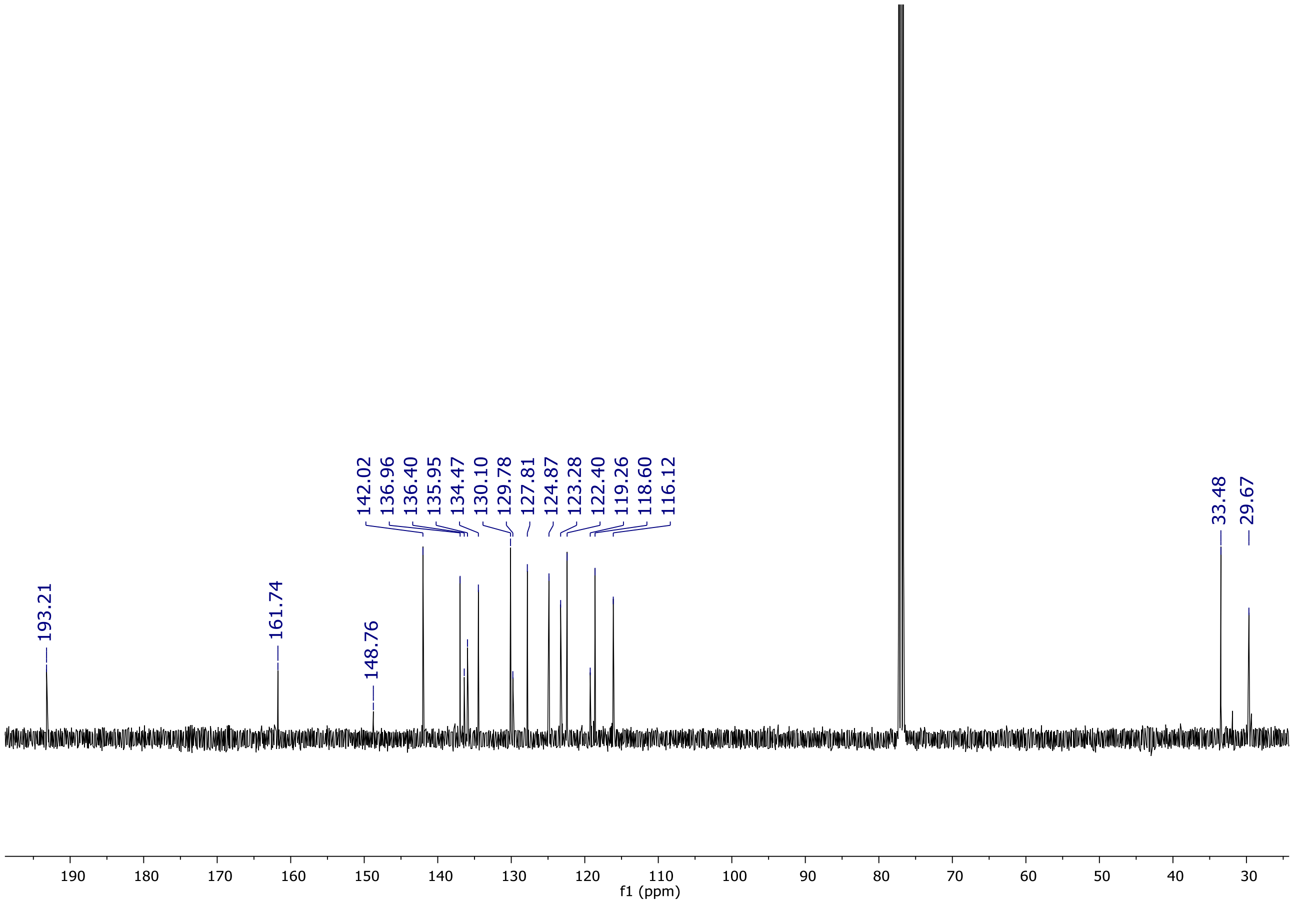
193.21
161.74
148.76
142.02
136.96
136.40
135.95
134.47
130.10
129.78
127.81
124.87
123.28
122.40
119.26
118.60
116.12
33.48
29.67
190 180 170 160 150 140 130 120 110 100 90 80 70 60 50 40 30
f1 (ppm)

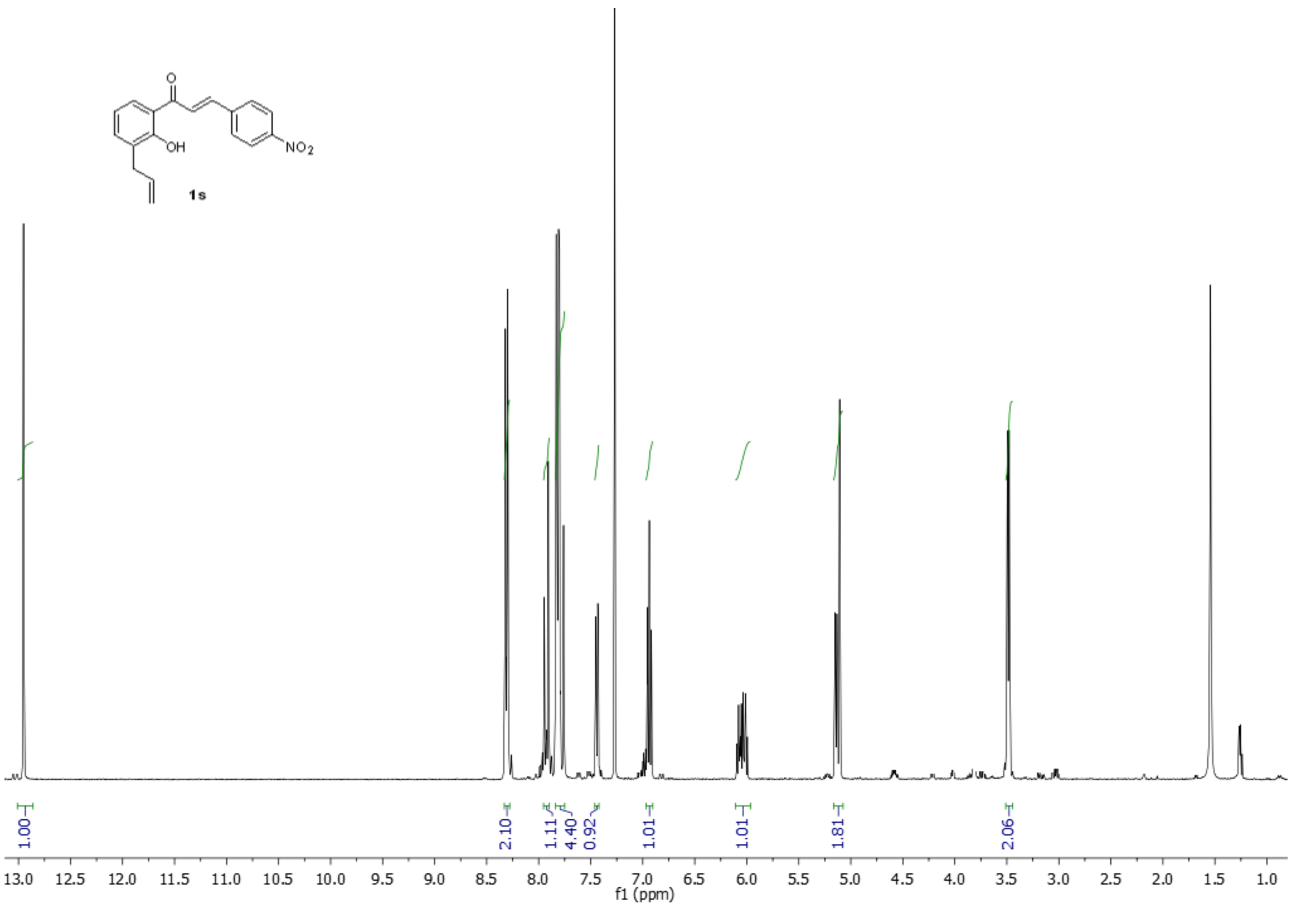
O
OH
NO2
1s
1.00
2.10
1.11
4.40
0.92
1.01
1.01
1.81
2.06
13.0 12.5 12.0 11.5 11.0 10.5 10.0 9.5 9.0 8.5 8.0 7.5 7.0 6.5 6.0 5.5 5.0 4.5 4.0 3.5 3.0 2.5 2.0 1.5 1.0
f1 (ppm)

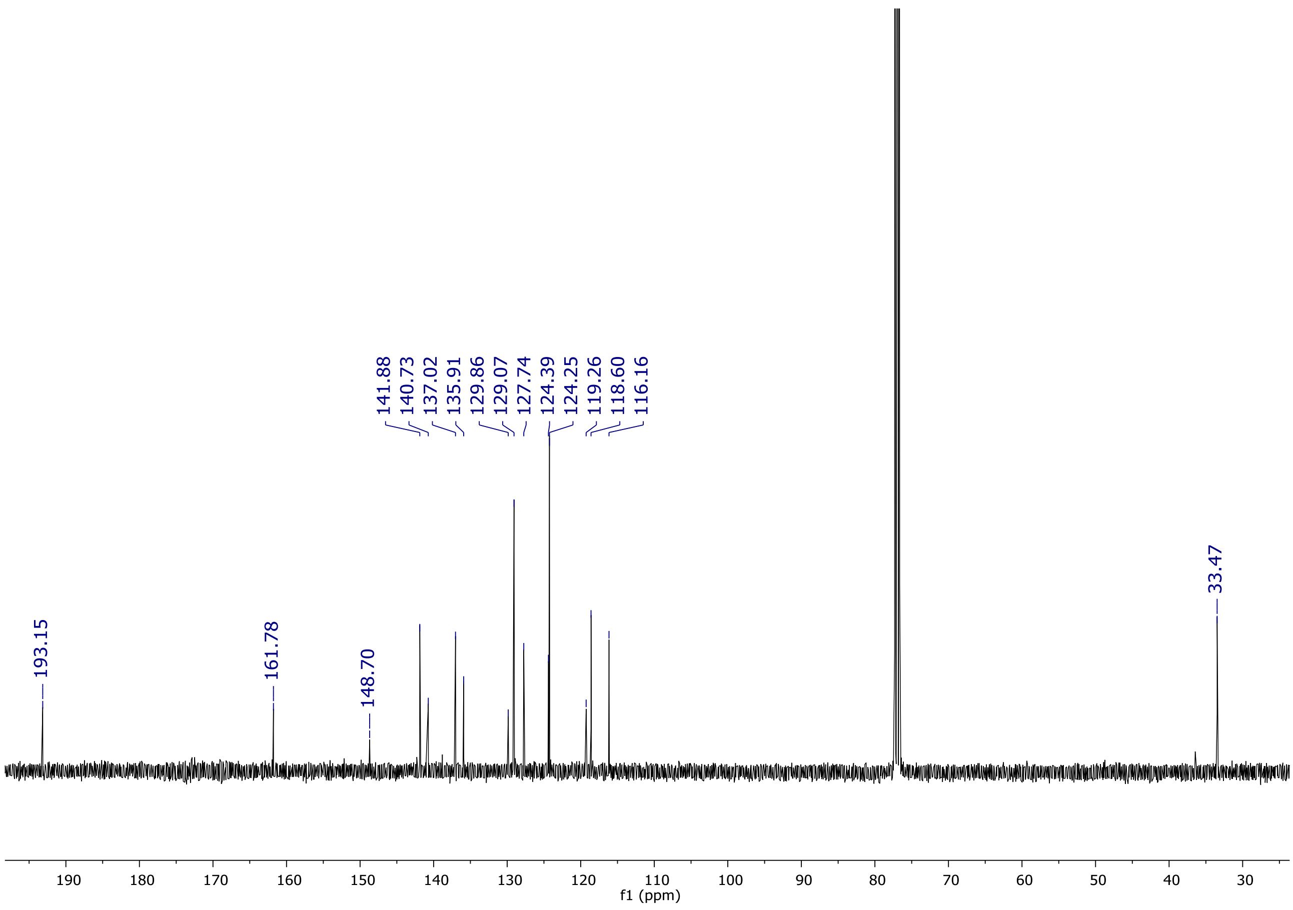
193.15
161.78
148.70
141.88
140.73
137.02
135.91
129.86
129.07
127.74
124.39
124.25
119.26
118.60
116.16
33.47
190 180 170 160 150 140 130 120 110 100 90 80 70 60 50 40 30
f1 (ppm)
